# Antimicrobial and Antiproliferative Properties of 2‐Phenyl‐*N*‐(Pyridin‐2‐yl)acetamides

**DOI:** 10.1111/cbdd.70030

**Published:** 2025-01-21

**Authors:** Daria Nawrot, Barbora Koutníková, Ondřej Janďourek, Klára Konečná, Martin Novák, Pavla Paterová, Pavel Bárta, Ghada Bouz, Jan Zitko, Martin Doležal

**Affiliations:** ^1^ Faculty of Pharmacy in Hradec Králové Charles University Hradec Králové Czech Republic; ^2^ Biomedical Research Centre University Hospital Hradec Kralove Hradec Králové Czech Republic; ^3^ Department of Clinical Microbiology University Hospital Hradec Králové Czech Republic

**Keywords:** antibacterial, antimycobacterial, antiproliferative, drug design, pyridine, tuberculosis

## Abstract

Infectious diseases, including bacterial, fungal, and viral, have once again gained urgency in the drug development pipeline after the recent COVID‐19 pandemic. Tuberculosis (TB) is an old infectious disease for which eradication has not yet been successful. Novel agents are required to have potential activity against both drug‐sensitive and drug‐resistant strains of *Mycobacterium tuberculosis* (*Mtb*), the causative agent of TB. In this study, we present a series of 2‐phenyl‐*N*‐(pyridin‐2‐yl)acetamides in an attempt to investigate their possible antimycobacterial activity, cytotoxicity on the HepG2 liver cancer cell line, and—as complementary testing—their antibacterial and antifungal properties against a panel of clinically important pathogens. This screening resulted in one compound with promising antimycobacterial activity—compound **12**, MIC_Mtb H37Ra_ = 15.625 μg/mL (56.26 μM). Compounds **17**, **24**, and **26** were further screened for their antiproliferative activity against human epithelial kidney cancer cell line A498, human prostate cancer cell line PC‐3, and human glioblastoma cell line U‐87MG, where they were found to possess interesting activity worth further exploration in the future.

## Graphical abstract

Among prepared 2‐phenyl‐*N*‐(pyridin‐2‐yl)acetamides, we identified compounds with promising in vitro antimycobacterial activity (notably compound **12**). In comparison with the previous series, the methylene‐extended linker improved antimycobacterial activity. Compound **26** was identified as a promising antiproliferative hit.

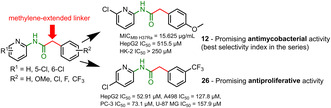

## 1 Introduction

Tuberculosis (TB), caused by *Mycobacterium tuberculosis* (*Mtb*), is a ubiquitous latent infection with about one fourth of the world's population being infected. As reported by the World Health Organization, 10.6 million people worldwide, mainly in Southeast Asia or South Africa, fell ill and 1.6 million people died of active tuberculosis in 2021 (World Health Organization 2022). Except for 2021, the year of COVID‐19 dominance, tuberculosis is globally the leading cause of death among infectious diseases. TB also remains the 13th leading cause of death and the primary cause of death in HIV‐positive individuals (World Health Organization 2022). A minority (5%–10%) with latent infection will develop active disease (World Health Organization 2022), depending on the status of health of the afflicted individual, among other factors. Usually, tuberculosis may be efficiently treated with first‐line drugs (pyrazinamide, rifampicin, ethambutol, and isoniazid) used in combinations. However, the treatment is long (the standard course of treatment is 6 months) and often causes a plethora of side effects, with hepatotoxicity being the most common and serious (Molla et al. 2021). An alarming issue, together with poor patient adherence, is antimicrobial resistance. Patients with resistant TB do not respond to first‐line treatment, and the alternative regimen used in such situations has more drawbacks, that is, a higher risk of hepatotoxicity, higher cost of the treatment, or lower treatment success rate (Araújo‐Mariz et al. 2016; Edwards et al. 2023; Lange et al. 2014).

**FIGURE 1:**
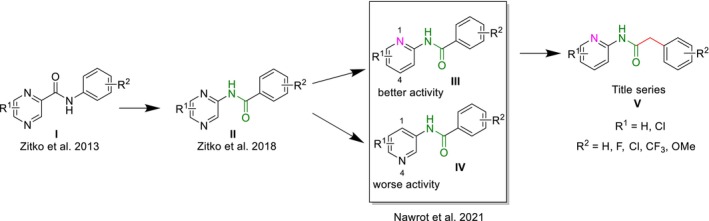
Design rationale of title compounds based on previously reported (Zitko et al. 2018, 2013; Nawrot et al. 2021) structure–activity (antimicrobial) relationships.

In the current work, and as a continuation of previous synthetic efforts of our research group, we report the design, synthesis, and biological evaluation of a series of 2‐phenyl‐*N*‐(pyridin‐2‐yl)acetamides. Our previous work allowed us to highlight the important structure–activity relationship toward antimicrobial activity in the group of binuclear heteroaromatic compounds with a carboxamidic linker (see Figure 1). Key observations from these studies include: (1) *N*‐pyrazinylbenzamides, Figure 1, structure (**II**) (Zitko et al. 2018) have a superior selectivity profile (lower toxicity) compared to *N*‐phenylpyrazine‐2‐carboxamides (**I**) (Zitko et al. 2013); (2) A nitrogen atom at position 1 of the pyridine ring of *N*‐pyridinylbenzamides is important for antimycobacterial activity (structures **III** vs. **IV**) (Nawrot et al. 2021); (3) R^2^ = CF_3_ substituent contributes to broadening the spectrum of antimycobacterial activity (Nawrot et al. 2021). Therefore, the main objectives of this work were to investigate the influence of extending the retroamide linker by inserting the methylene group (**V**) and the influence of various substituents R^1^ and R^2^ (especially different positions of the ‐CF_3_ substituent, since in our previous work we explored 3‐CF_3_ only) on the antimycobacterial and cytotoxic activities. All compounds were screened against *Mycobacterium tuberculosis* H37Ra, *Mycolicibacterium aurum, Mycolicibacterium smegmatis*, (with additional testing on *Mycobacterium tuberculosis* H37Rv, *M. avium* , and *M. kansasii* ), against a panel of bacteria ( *Staphylococcus aureus* [ *S. aureus*], methicillin‐resistant *S. aureus* , *Escherichia coli* , *Pseudomonas aeruginosa* , *Acinetobacter baumannii* , *Staphylococcus epidermidis* , *Enterococcus faecalis* , *Klebsiella pneumoniae*) *an*d a panel of fungi ( *Candida albicans* , *Candida krusei* , *Candida parapsilosis* , *Candida tropicalis* , *Aspergillus fumigatus*, *Aspergillus flavus*, *Lichtheimia corymbifera*, and *Trichophyton interdigitale*). Selected compounds were tested for their hepatotoxicity on the HepG2 cell line (with additional testing on cancer cell lines A498, PC‐3 and U‐87 MG an normal cell line HK‐2). A literature search revealed that none of the title compounds were previously investigated for biological activity except for compound **10**, which was investigated as a hPPAR (human peroxisome proliferator‐activated receptor) activator for treating diabetes and cardiovascular diseases (Cadilla et al. 2003).

## 2 Materials and Methods

### 2.1 General Information

All reagents and solvents (unless stated otherwise) were purchased from Merck (Darmstadt, Germany) and used without further purification. Reaction progress and purity of products were monitored using Silica 60 F_254_ TLC plates (Merck, Darmstadt, Germany). Flash chromatography of the final compounds was performed on a puriFlash XS420+ (Interchim, Montluçon, France) with original columns (silica, 30 μm) provided by the same company. The mobile phase was gradient 0%–50% ethyl acetate (EtOAc) in hexane (Hex), and detection was performed by UV–VIS detector at wavelengths of 254 nm and 280 nm.

The NMR spectra of **1–3**, **6**, **7–12**, **18**, **19**, **21**, **23**, **31**, and **32**, were recorded on a Varian VNMR S500 (Varian, Palo Alto, CA, USA) at 500 MHz for ^1^H and 126 MHz for ^13^C. The spectra of the remaining compounds were measured on Jeol JNM‐ECZ600R at 600 MHz for ^1^H and 151 MHz for ^13^C. The spectra were recorded in DMSO‐*d*_6_ or CDCl_3_ at ambient temperature. The chemical shifts are reported as δ values in ppm and are indirectly referenced to tetramethylsilane (TMS) via the solvent signal (2.49 for ^1^H and 39.7 for ^13^C in DMSO‐*d*_6_; 7.27 for ^1^H and 77.0 for ^13^C in CDCl_3_). IR spectra were recorded on a NICOLET 6700 FT‐IR spectrophotometer (Nicolet, Madison, WI, USA) using the ATR‐Ge method. Elemental analysis was done on a Vario MICRO cube Elemental CHNS Analyzer (Elementar Analysensysteme, Hanau, Germany) with values given as a percentage. Yields are given in percent and refer to the amount of pure product after all purification steps. Log*P* values were calculated using ChemDraw v20.1. (PerkinElmer Informatics, Waltham, MA, USA).

### 2.2 Chemistry

#### 2.2.1 Synthesis

Three different procedures were used to prepare the title compounds: 1,1′‐carbonyldiimidazole (CDI) coupling (procedure A), activation of acid by oxalyl chloride (procedure B, when synthesis using CDI did not produce final compounds in reasonable yields) and direct reaction of commercially obtained phenylacetyl chloride with the corresponding amine (procedure C).

Compounds **4, 5, 13**–**17, 22, 25, 28, 29** were prepared by procedure A; compounds **1**, **2**, **3**, **6, 18, 23, 24, 26, 27**, and **30** by procedure B, while compounds **7**–**12, 19**–**21**, and **31**–**33** by procedure C.

##### 2.2.1.1 Procedure A

The corresponding phenylacetic acid (2 mmol) was placed in a 50 mL round‐bottom flask, 2.2 mmol of 1,1′‐carbonyldiimidazole (CDI) was added, and the mixed solids were heated with a heat gun for 30 s. The sample was placed on a stirring plate, and 10 mL of anhydrous dichloromethane (DCM) was added (heavy bubbling of CO_2_ appeared). The sample was heated and stirred for 30 min, and then 2 mmol of the corresponding amine dissolved in 5 mL of DCM was added. The mixture was stirred overnight at room temperature (RT). The workup and purification process are described below (Section Purification).

##### 2.2.1.2 Procedure B

The corresponding phenylacetic acid (2 mmol) was placed in a 50 mL round‐bottom flask, 15 mL of anhydrous DCM was added, and the dispersion was stirred. In a separate 50 mL Erlenmeyer flask, 2 mmol (254 mg) of oxalyl chloride was added to 10 mL of anhydrous DCM. The content of the Erlenmeyer flask was transferred to the flask with the phenylacetic acid and three drops of *N*,*N*‐dimethylformamide (DMF) were added (mixture 1). Heavy bubbling appeared. The flask was closed with a stopper and stirred for another 30 min. Another 100 mL round‐bottom flask with 10 mL of DCM was placed in an ice bath, and 2 mmol of the corresponding amine was added, along with 4.5 mmol of anhydrous pyridine (mixture 2). After mixture 2 cooled down, mixture 1 was added dropwise with stirring. The flask was closed with a stopper, stirred for 20 min, taken out from the ice bath, and stirred. The progress of the reaction was monitored by TLC (silica gel plates, Hex:EtOAc, 2:1). After 3 h, the reaction was complete (no further changes visible on the TLC plate). The reaction mixture was transferred into a separatory funnel and washed with distilled water (2–3 times, 30 mL). The combined organic layers were dried over anhydrous sodium sulfate, filtered, and subjected to the purification process described below (Section Purification).

##### 2.2.1.3 Procedure C

The corresponding amine (2 mmol) was placed in a 50 mL round‐bottom flask, and 10 mL of anhydrous DCM and anhydrous pyridine (3 mmol, 237 mg) were added. The flask was closed with a stopper and cooled in an ice bath for 20 min. Selected phenylacetyl chloride (2.4 mmol) was diluted in 3 mL of anhydrous DCM and added dropwise upon stirring to the mixture, the flask was closed with a stopper and stirred at RT. The progress of the reaction was monitored by TLC (silica gel plates, Hex:EtOAc, 2:1). After 3 h, the reaction was complete (no further changes observed on the TLC plate). The reaction mixture was washed with distilled water (2–3 times, 30 mL). The combined organic layers were dried over anhydrous sodium sulfate, filtered, and subjected to the purification process described below (Section Purification).

#### 2.2.2 Purification

Upon completion, the reaction mixture was adsorbed on silica by evaporating the solvent under reduced pressure, placed into a solid loading cartridge, and submitted to flash chromatography (silica column 30 g, gradient elution 0%–50% EtOAc in Hex, flow rate 20 mL/min, detection wavelength 254 and 280 nm). Fractions containing pure product were collected and combined, and solvents were evaporated under reduced pressure to yield oily liquids, which, after some time, solidified at room temperature.

### 2.3 Evaluation of Biological Activity

The methodology used in this publication is consistent with our previous publications. A full description of the methods can be found in Data S1.

#### 2.3.1 In Vitro Antimycobacterial Activity

Testing was performed by Microplate Alamar Blue Assay (MABA) (Franzblau et al. 1998) and according to EUCAST protocol (Schön et al. 2020), where results were expressed as MIC in μg/mL in comparison with INH, RIF, and ciprofloxacin (CIP) as standards.

#### 2.3.2 In Vitro Antibacterial Activity

Microdilution broth method according to EUCAST with minor modifications (European Committee for Antimicrobial Susceptibility Testing [EUCAST] of the European Society of Clinical Microbiology and Infectious Diseases [ESCMID] 2003). Results were expressed as MIC compared to standards of gentamicin (GNT) and ciprofloxacin (CIP).

#### 2.3.3 In Vitro Antifungal Activity

A microdilution broth method was performed according to EUCAST protocols with minor modifications (EUCAST 2020a, 2020b). Results were expressed as MIC compared to standards amphotericin B (AMB) and voriconazole (VRC).

#### 2.3.4 In Vitro Cytotoxicity Determination

The cytotoxicity of final compounds was determined in human hepatocellular carcinoma cell line HepG2. Additional testing of selected compounds was performed on human epithelial kidney cancer cell line A498, human prostate cancer cell line PC‐3, human glioblastoma cell line U‐87 MG, and normal human proximal tubule cell line HK‐2. To measure the viability CellTiter 96 AQueous One Solution Cell Proliferation Assay (CellTiter 96; PROMEGA, Fitchburg, USA) was used, and the results were expressed as inhibitory concentration which reduces viability of the cell population to 50% from the maximum viability (IC_50_) and calculated by nonlinear regression from a semi‐logarithmic plot of incubation concentration versus percentage of the absorbance relative to untreated controls.

## 3 Results and Discussion

### 3.1 Synthesis and Characterization

Presented title compounds were prepared by three different methods; either by direct reaction between amine (2‐aminopyridine, 5‐chloropyridin‐2‐amine, and 6‐chloropyridin‐2‐amine) and corresponding phenylacetyl chloride or by activation of substituted phenylacetic acid (coupling with CDI or reaction of acid with oxalyl chloride) and further reaction with corresponding amine.

Reactions employing phenylacetyl chlorides were performed in the presence of dry pyridine as a base. Moreover, pyridine was used to prevent the formation of diacetylated products, which were often observed in reactions with oxalyl chloride. Since this type of reaction is also exothermic, the solution of amine and pyridine in anhydrous DCM was cooled in an ice bath before, during, and after the addition of phenylacetyl chloride.

Pure products were obtained after flash column chromatography on silica, using hexane and ethyl acetate as mobile phase. Compounds were characterized by NMR spectra (^1^H and ^13^C), elemental analysis, melting points, and IR. The obtained data were consistent with the proposed structures. In the ^1^H NMR spectra, the amidic hydrogen signal appeared as a singlet at 10.40–11.06 ppm (in DMSO‐*d*_*6*_) or 7.90–8.37 ppm (in CDCl_3_). In the ^13^C NMR spectra, the amidic carbon signal appeared at 169.17–170.78 ppm (in DMSO‐*d*_*6*_) or 168.94–170.01 ppm (in CDCl_3_). After all purification steps, the final compounds were isolated as solids (of white to pale yellow color) in moderate yields, ranging from 11% to 92%. The full analytical data of prepared compounds are located in Data S1, section 1.7.

Compounds **12** and **24** were incubated in phosphate‐buffered saline (pH 7.4) at 37°C for 180 min to assess their hydrolytic stability in the context of the present amidic bond. Both compounds proved stable as their concentration did not decline below 90% during the experiment. For methodology and stability charts, see Data S1.

### 3.2 Biological Activity

#### 3.2.1 Antimycobacterial Activity

All synthesized compounds were screened for their activity against *Mtb* H37Ra (an avirulent strain commonly used as a surrogate model for *Mtb* H37Rv), *M. smegmatis*, and *M. aurum* . Sixteen out of a total of 33 compounds had activity against *Mtb* H37Ra (strain of main interest) equal to or below 31.25 μg/mL (cutoff value for activity). The lowest value of MIC = 7.81 μg/mL was recorded for three compounds, namely **20** (R^1^ = 5‐Cl, R^2^ = 4‐Cl), **21** (R^1^ = 6‐Cl, R^2^ = 4‐Cl), and **30** (R^1^ = 6‐Cl, R^2^ = 4‐CF_3_), refer to Table 1. The latter compounds were advanced into testing against the virulent strain *Mtb* H37Rv, where they were found to preserve their antimycobacterial activity (MIC = 6.25 μg/mL), *M. avium* , and *M. kansasii* (Table 2). Five other compounds (**2**, **12**, **18**, **32**, and **33**) had lower but still promising activity on Mtb H37Ra (MIC = 15.625 μg/mL). For all compounds with activity on Mtb H37Ra below the cutoff value (≤ 31.25 μg/mL), we calculated the selectivity index, which indicated that the most effective compound based on the window between cytotoxic effect and antimycobacterial activity is compound **12**.

**TABLE 1:** Title compounds, their antimycobacterial activity (expressed as minimum inhibitory concentration, MIC), and cytotoxicity on the HepG2 cell line (expressed as IC_50_).

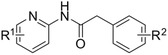									
No	R^1^	R^2^	log*P* ^*a*^	*Mtb* H37Ra (μg/mL)	*Mtb* H37Ra (μM)^b^	SI	*M. smegmatis* (μg/mL)	*M. aurum* (μg/mL)	HepG2 IC_50_ (μM)
1	H	H	2.16	31.25	147.24	6.8	62.5	250	> 1000^d^
2	5‐Cl	H	2.72	15.625	63.34	> 1.6	500	500	> 100^c^
3	6‐Cl	H	3.06	31.25	126.68	3.42	15.625	62.5	433.2
4	H	2‐OMe	2.04	125	515.98		> 500	250	> 1000^d^
5	5‐Cl	2‐OMe	2.59	> 500	> 1800.45		> 500	> 500	> 250^c^
6	6‐Cl	2‐OMe	2.94	> 500	> 1800.45		> 500	> 500	> 500^c^
7	H	3‐OMe	2.04	125	515.98		250	250	> 500^c^
8	5‐Cl	3‐OMe	2.59	31.25	112.53	1.4	> 500	> 500	157.8
9	6‐Cl	3‐OMe	2.94	250	900.22		125	> 500	638.5
10	H	4‐OMe	2.04	125	515.98		62.5	250	> 1000^d^
11	5‐Cl	4‐OMe	2.59	> 500	> 1800.45		> 500	> 500	399.0
12	6‐Cl	4‐OMe	2.94	15.625	56.26	9.2	62.5	62.5	515.5
13	H	2‐Cl	2.72	62.5	253.37		> 500	250	754.7
14	5‐Cl	2‐Cl	3.28	> 500	> 1778.57		> 500	> 500	> 50^c^
15	6‐Cl	2‐Cl	3.62	31.25	111.16	> 0.9	> 500	250	> 100^c^
16	H	3‐Cl	2.72	62.5	253.37		> 500	250	> 250^c^
17	5‐Cl	3‐Cl	3.28	125	444.64		> 500	250	112.5
18	6‐Cl	3‐Cl	3.62	15.625	55.58	> 1.8	> 500	62.5	> 100^c^
19	H	4‐Cl	2.72	31.25	126.68	2.6	31.25	> 500	333.1
20	5‐Cl	4‐Cl	3.28	7.81	27.78	1.7	500	500	48.5
21	6‐Cl	4‐Cl	3.62	7.81	27.78	2.9	15.625	31.25	81.5
22	H	2‐CF_3_	3.08	125	446.06		250	250	445.3
23	5‐Cl	2‐CF_3_	3.64	31.25	99.31	2.0	> 500	> 500	200.6
24	6‐Cl	2‐CF_3_	3.98	> 500	> 1588.94		> 500	> 500	64.58
25	H	3‐CF_3_	3.08	62.5	223.03		> 500	125	724.1
26	5‐Cl	3‐CF_3_	3.64	> 500	> 1588.94		125	> 500	52.91
27	6‐Cl	3‐CF_3_	3.98	31.25	99.31	1.5	> 500	15.625	146.5
28	H	4‐CF_3_	3.08	31.25	111.52	3.7	> 500	> 500	408.2
29	5‐Cl	4‐CF_3_	3.64	125	397.23		> 500	> 500	> 50^c^
30	6‐Cl	4‐CF_3_	3.98	7.81	24.82	3.7	> 500	> 500	92.6
31	H	4‐F	2.32	125	542.95		62.5	250	516.9
32	5‐Cl	4‐F	2.88	15.625	59.04	4.2	62.5	500	> 250^c^
33	6‐Cl	4‐F	3.22	15.625	59.04	3.6	500	250	213
INH			−0.64	0.125–0.25	0.9–1.8		7.81–15.625	3.91–7.81	NA
RIF			2.70	0.003125	0.0037		1.56–6.25	0.39–0.78	NA
CIP			1.32	0.125–0.25	0.38–0.75		0.125–0.0625	0.015625‐0.03125	NA

Abbreviations: CIP, ciprofloxacin; INH, isoniazid; NA, not available; RIF, rifampicin.

^a^ Calculated in ChemDraw v20.

^b^ Calculated from MIC (μg/mL).

^c^ Compound precipitated in the testing medium.

^d^ HepG2 IC_50_ values were over the limit of the tested concentrations. SI –selectivity index = HepG2 IC_50_ (μM)/MIC_*Mtb*H37Ra_ (μM), calculated for compounds with MIC ≤ 31.25 μg/mL.

**TABLE 2:** Comparison of biological activity profiles of compounds **20**, **21**, and **30**.

	Compound 20 (R^1^ = 5‐Cl, R^2^ = 4‐Cl)	Compound 21 (R^1^ = 6‐Cl, R^2^ = 4‐Cl)	Compound 30 (R^1^ = 6‐Cl, R^2^ = 4‐CF_3_)
MIC_*Mtb* H37Ra_	7.81 μg/mL	7.81 μg/mL	7.81 μg/mL
MIC _*M. smegmatis*_	500 μg/mL	15.625 μg/mL	> 500 μg/mL
MIC _*M. aurum*_	500 μg/mL	31.25 μg/mL	> 500 μg/mL
MIC _*M. kansasii*_	> 100 μg/mL	25 μg/mL	> 100 μg/mL
MIC _*M. avium*_	> 100 μg/mL	6.25 μg/mL	6.25 μg/mL
MIC_Mtb H37Rv_	6.25 μg/mL	6.25 μg/mL	6.25 μg/mL
Antibacterial activity	None	250 μM on SA, 250 μM on MRSA	None
Antifungal activity	None	62.5 μM on TI	None
HepG2 Cytotoxicity	IC_50_ = 48.5 μM	IC_50_ = 81.5 μM	IC_50_ = 92.6 μM

Abbreviations: MRSA, methicillin resistant *Staphylococcus aureus* ; SA, *Staphylococcus aureus* ; TI, *Trichophyton interdigitale*.

Regarding structure–activity relationships, when it comes to the substitution R^1^ on the pyridine ring (H, 5‐Cl, and 6‐Cl), it is apparent from Table 1 that having chlorine at position 6 favors antimycobacterial activity, while compounds with an unsubstituted pyridine ring were generally less active. This conclusion agrees with previous results (Nawrot et al. 2021). The influence of the position of Cl on the pyridine ring was most apparent when R^2^ is 4‐Cl. In general, the most favorable substitution on the benzene ring was R^2^ = 4‐Cl (compounds **20** and **21** with MIC_Mtb H37Ra_ = 7.81 μg/mL). Table 2 shows a direct comparison between compounds **20** and **21**, differing in the position of chlorine on the pyridine ring while they both bear R^2^ = 4‐Cl. By changing chlorine from position six to position five (R^1^), activity on *M. smegmatis* , *M. aurum*, *M.* kansasii, and *M. avium* was lost. Also, a loss of modest antibacterial and antifungal activity was observed. Not only did the 6‐Cl derivative (**21**) have a broader spectrum of activity, but it was also twofold less cytotoxic compared to **20**.

**FIGURE 2:**
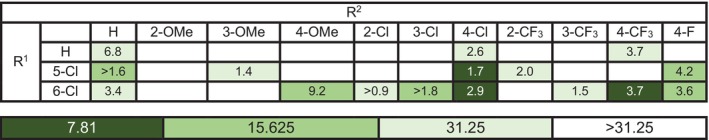
The influence of R^1^ and R^2^ substitution on antimycobacterial activity. Color coding denotes antimycobacterial activity against *Mtb* H37Ra (MIC in μg/mL), darker means more active; numbers indicate the selectivity index SI = HepG2 IC_50_ (μM)/MIC_*Mtb*H37Ra_ (μM), calculated for compounds with MIC ≤ 31.25 μg/mL.

We also found that the ‐CF_3_ substituent still favors activity, as in previous publication (Nawrot et al. 2021), and the position of substitution affects the level of activity, with 4‐CF_3_ being the most active among others (compounds **30**). A R^2^ substitution in position 2 was not favorable whether the substituent was lipophilic (R^2^ = 2‐Cl or 2‐CF_3_) or more hydrophilic (R^2^ = 2‐OMe). Figure 2 demonstrates the influence of different substituents at positions R^1^ and R^2^ on antimycobacterial activity (*Mtb* H37Ra) with corresponding selectivity indices.

Aside from *Mtb* H37Ra and H37Rv, only three compounds **3**, **21**, and **27** showed moderate activity (MIC = 15.625 μg/mL) on *M. aurum* or *M. smegmatis* (see Table 1), while compound **21** showed additional activity on *M. kansasii* and *M. avium* (see Table 2). Compound **30** was active against *M. avium* .

Compared to the previously published series (Nawrot et al. 2021), the elongated linker influenced antimycobacterial activity in the following way: In 10 matching pairs, title compounds had better activity, in 5 pairs the activity was equal, and in 9 pairs, the shorter linker compounds had better activity. Interestingly, the effect of linker elongation was most visible in R^2^ = H, 4‐Cl and 3‐CF_3_. When R^2^ was unsubstituted (R^2^ = H) or substituted by chlorine in position 4, the title compounds exhibited better antimycobacterial activity compared to the previously published counterparts. The opposite was found in all three 3‐CF_3_ substituted compounds, where the linker elongation decreased their antimycobacterial effect. The most dramatic decrease in activity was observed for compounds **5**, **11**, and **26**, refer to Table 3.

**TABLE 3:** Comparison of antimycobacterial activity against *Mtb* H37Ra of title series and previously published *N*‐pyridinyl benzamides.

		Title series			Previously published compounds (Nawrot et al. 2021)
		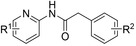			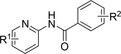
R^1^	R^2^	Code	MIC *Mtb* H37Ra (μg/mL)	Relative activity^a^	MIC *Mtb* H37Ra (μg/mL)
H	H	1	31.25	>	250
5‐Cl	H	2	15.625	>	> 500
6‐Cl	H	3	31.25	>	125
H	2‐Cl	13	62.5	>	> 500
5‐Cl	2‐Cl	14	> 500	=	> 500
6‐Cl	2‐Cl	15	31.25	>	62.5
H	3‐Cl	16	62.5	=	62.5
5‐Cl	3‐Cl	17	125	<	31.25
6‐Cl	3‐Cl	18	15.625	=	15.625
H	4‐Cl	19	31.25	>	62.5
5‐Cl	4‐Cl	20	7.81	>	> 500
6‐Cl	4‐Cl	21	7.81	>	> 500
H	2‐OMe	4	125	<	31.25
5‐Cl	2‐OMe	5	> 500	<	31.25
6‐Cl	2‐OMe	6	> 500	=	> 500
H	3‐OMe	7	125	<	62.5
5‐Cl	3‐OMe	8	31.25	>	62.5
6‐Cl	3‐OMe	9	250	<	31.25
H	4‐OMe	10	125	<	62.5
5‐Cl	4‐OMe	11	> 500	<	62.5
6‐Cl	4‐OMe	12	15.625	=	15.625
H	3‐CF_3_	25	62.5	<	31.25
5‐Cl	3‐CF_3_	26	> 500	<	7.81
6‐Cl	3‐CF_3_	27	31.25	<	7.81

^a^ Related to 1/MIC.

#### 3.2.2 Antibacterial and Antifungal Activities

None of the tested compounds exerted significant antibacterial or antifungal activities. Tables with full results can be found in the Data S1. The reason for the absence of antibacterial activity in the current series is unknown. However, the preference for antimycobacterial activity over general antibacterial activity was noted in all previously published, structurally related series (Zitko et al. 2018, 2013; Nawrot et al. 2021) mentioned in the Introduction Figure 1. The exception was *N*‐pyrazinylhydroxybenzamides (Kerda et al. 2023), which were reported to have significant antistaphylococcal activity. In these derivatives, however, the activity was bound to the 2‐OH or 4‐OH substitution on the phenyl ring, the former probably due to the resemblance to salicylanilides. In our current series, there are no derivatives with such substituents.

#### 3.2.3 Cytotoxicity

All synthesized compounds were screened in vitro for their cytotoxic activity on hepatocellular carcinoma cells (HepG2). Results showed that 9 out of 33 compounds have an IC_50_ value higher than 500 μM, and two of them had good activity in vitro against mycobacteria (**1** and **12**). Obtained IC_50_ values were used to calculate selectivity indices (SI) as the ratio of the IC_50_ value against HepG2 to the MIC value against *Mtb* H37Ra. Ten compounds had solubility issues and precipitated in the testing medium, making the determination of their exact IC_50_ and accurate SI values not feasible. Among all, compound **12** had the highest SI value of 9.2 and therefore is the most promising compound as an antimycobacterial agent (Table 1). Notably, the cytotoxicity of **12** was also low in noncancerous human proximal tubule cell line HK‐2 (IC_50_ > 250 μM).

Where possible (the compound did not precipitate in the testing medium), we inspected the influence of R^1^ position and/or the nature of the substituent on cytotoxicity. In three out of four compared groups (R^2^ = 4‐OMe, 4‐Cl, and 3‐CF_3_), the lowest IC_50_ values were found for R^1^ = 5‐Cl. In a group where R^2^ = 2‐CF_3_, the lowest IC_50_ values were found for R^1^ = 6‐Cl. In general, for all groups, derivatives with non‐chlorinated pyridine (R^1^ = H) were the least cytotoxic against the HepG2 cell line.

Cytotoxicity testing combined with antimicrobial screening allowed us to select three compounds (**17**, **24**, and **26**) based on their selectivity profiles (no or low antimicrobial activity, with low IC_50_ value against the HepG2 line and good solubility in testing medium) to further explore their potential as antiproliferative/anticancer agents. These three compounds were tested against human epithelial kidney cancer cell line A498, human glioblastoma cell line U‐87 MG, prostate cancer cell line PC‐3, and noncancerous human proximal tubule cell line HK‐2. Based on the results shown in Table 4, the three compounds showed cytotoxic activities against all the tested cell lines, suggesting their potential as a starting point for further optimization to develop new anticancer compounds. The cytotoxic effect was not selective toward cancerous cell lines, but this cannot be reasonably expected at this early development stage.

**TABLE 4:** Cytotoxicity profile of selected compounds.

	Structure		IC_50_ [μM]				
No.	R^1^	R^2^	HepG2	A498	PC‐3	U‐87 MG	HK‐2
**12**	6‐Cl	4‐OMe	515.5	n.a.	n.a.	n.a.	> 250
**17**	5‐Cl	3‐Cl	112.5	135	212.7	161.1	137.5
**24**	6‐Cl	2‐CF_3_	64.58	278.4	> 100	> 250	247.3
**26**	5‐Cl	3‐CF_3_	52.91	127.8	73.1	157.9	29.9
	Tamoxifen		19.6	52.4	5.4	21.6	n.a.

Abbreviations: A498, human epithelial kidney carcinoma; HepG2, human hepatocellular liver carcinoma; HK‐2, human kidney proximal tubule (normal cell line); n.a., not available; PC‐3, human prostate adenocarcinoma; U‐87 MG, human glioblastoma astrocytoma.

## 4 Conclusions

As an attempt to develop novel, potentially active antimycobacterials, and in continuation of our previous work, we report the synthesis and biological evaluation of a series of 2‐phenyl‐*N*‐(pyridin‐2‐yl)acetamides. Out of 33 synthesized compounds, compound **12** (R^1^ = 6‐Cl; R^2^ = 4‐OMe) was the most promising antimycobacterial candidate with significant activity MIC_Mtb H37Ra_ = 15.625 μg/mL (56.26 μM) and low cytotoxicity (HepG2 IC_50_ = 515.5 μM, HK‐2 IC_50_ > 250 μM) and could be further investigated as an antimycobacterial agent. The mechanism of action (molecular target) remains to be determined. Compounds **17**, **24**, and **26** were investigated as potential anticancer agents since they possessed no or negligible antimicrobial activity but exhibited cytotoxicity on the HepG2 cell line. Their activity on A498, PC‐3, and U‐87 MG cancer cell lines showed they can be developed further in this direction. The mechanism of their nonspecific cytotoxic effect must be the subject of future investigation.

## Conflicts of Interest

The authors declare no conflicts of interest.

## Supporting information


**Data S1.** Biological assays—methods and full results, analytical data of prepared compounds, and representative NMR spectra in graphic form.

## Data Availability

The data that supports the findings of this study are available in the Data S1 of this article.

## References

[cbdd70030-bib-0001] Araújo‐Mariz, C. , E. P. Lopes , B. Acioli‐Santos , et al. 2016. “Hepatotoxicity During Treatment for Tuberculosis in People Living With HIV/AIDS.” PLoS One 11, no. 6: e0157725. 10.1371/journal.pone.0157725. 27332812 PMC4917242

[cbdd70030-bib-0002] Cadilla, R. H. , B. Richard , M. H. Lambert III , G. K. Liu , and J. S. Smith . 2003. “Preparation of Phenoxyalkanoic Acid Derivatives as hPPAR Activators for Treatment of Diabetes and Cardiovascular Diseases.” WO2003074495 (WO2003‐US5953).

[cbdd70030-bib-0003] Edwards, B. D. , H. Mah , N. F. Sabur , and S. K. Brode . 2023. “Hepatotoxicity and Tuberculosis Treatment Outcomes in Chronic Liver Disease.” Journal of the Association of Medical Microbiology and Infectious Disease Canada 8, no. 1: 64–74. 10.3138/jammi-2022-0029. 37008589 PMC10052910

[cbdd70030-bib-0004] EUCAST . 2020a. “Definitive Document E.DEF 7.3.2. Method for the Determination of Broth Dilution Minimum Inhibitory Concentrations of Antifungal Agents for Yeasts.” https://www.eucast.org/fileadmin/src/media/PDFs/EUCAST_files/AFST/Files/EUCAST_E_Def_7.3.2_Yeast_testing_definitive_revised_2020.pdf. 10.1111/j.1469-0691.2012.03880.x 22563750

[cbdd70030-bib-0005] EUCAST . 2020b. “Definitive Document E.DEF 9.3.2. Method for the Determination of Broth Dilution Minimum Inhibitory Concentrations of Antifungal Agents for Conidia Forming Moulds.” https://www.eucast.org/fileadmin/src/media/PDFs/EUCAST_files/AFST/Files/EUCAST_E_Def_9.3.2_Mould_testing_definitive_revised_2020.pdf. 10.1111/j.1469-0691.2008.02086.x 18828858

[cbdd70030-bib-0006] European Committee for Antimicrobial Susceptibility Testing (EUCAST) of the European Society of Clinical Microbiology and Infectious Diseases (ESCMID) . 2003. “Determination of Minimum Inhibitory Concentrations (MICs) of Antibacterial Agents by Broth Dilution.” Clinical Microbiology and Infection 9, no. 8: ix–xv. 10.1046/j.1469-0691.2003.00790.x.

[cbdd70030-bib-0007] Franzblau, S. G. , R. S. Witzig , J. C. McLaughlin , et al. 1998. “Rapid, Low‐Technology MIC Determination With Clinical *Mycobacterium tuberculosis* Isolates by Using the Microplate Alamar Blue Assay.” Journal of Clinical Microbiology 36, no. 2: 362–366. 10.1128/JCM.36.2.362-366.1998. 9466742 PMC104543

[cbdd70030-bib-0008] Kerda, M. , P. Šlechta , O. Jand'ourek , et al. 2023. “ *N*‐Pyrazinylhydroxybenzamides as Biologically Active Compounds: A Hit‐Expansion Study and Antimicrobial Evaluation.” Future Medicinal Chemistry 15: 1791–1806. 10.4155/fmc-2023-0189. 37877255

[cbdd70030-bib-0009] Lange, C. , I. Abubakar , J. W. C. Alffenaar , et al. 2014. “Management of Patients With Multidrug‐Resistant/Extensively Drug‐Resistant Tuberculosis in Europe: A TBNET Consensus Statement.” European Respiratory Journal 44, no. 1: 23–63. 10.1183/09031936.00188313. 24659544 PMC4076529

[cbdd70030-bib-0010] Molla, Y. , M. Wubetu , and B. Dessie . 2021. “Anti‐Tuberculosis Drug Induced Hepatotoxicity and Associated Factors Among Tuberculosis Patients at Selected Hospitals, Ethiopia.” Hepatic Medicine: Evidence and Research 13: 1–8. 10.2147/HMER.S290542. 33536799 PMC7850419

[cbdd70030-bib-0011] Nawrot, D. , E. Suchánková , O. Janďourek , et al. 2021. “N‐Pyridinylbenzamides: An Isosteric Approach Towards New Antimycobacterial Compounds.” Chemical Biology & Drug Design 97, no. 3: 686–700. 10.1111/cbdd.13804. 33068457

[cbdd70030-bib-0012] Schön, T. , J. Werngren , D. Machado , et al. 2020. “Antimicrobial Susceptibility Testing of *Mycobacterium tuberculosis* Complex Isolates—The EUCAST Broth Microdilution Reference Method for MIC Determination.” Clinical Microbiology and Infection 26, no. 11: 1488–1492. 10.1016/j.cmi.2020.07.036. 32750539

[cbdd70030-bib-0013] World Health Organization . 2022. Global Tuberculosis Report, 2023. Geneve, Switzerland: WHO.

[cbdd70030-bib-0014] Zitko, J. , A. Mindlová , O. Valášek , et al. 2018. “Design, Synthesis and Evaluation of *N*‐Pyrazinylbenzamides as Potential Antimycobacterial Agents.” Molecules 23, no. 9: 2390. 10.3390/molecules23092390. 30231544 PMC6225228

[cbdd70030-bib-0015] Zitko, J. , B. Servusová , P. Paterová , et al. 2013. “Synthesis, Antimycobacterial Activity and in Vitro Cytotoxicity of 5‐Chloro‐*N*‐Phenylpyrazine‐2‐Carboxamides.” Molecules 18, no. 12: 14807–14825. 10.3390/molecules181214807. 24317522 PMC6270209

