# Supplementary material for: Antimicrobial and Antiproliferative Properties of 2‐Phenyl‐N‐(Pyridin‐2‐yl)acetamides
Source: Chem Biol Drug Des. 2025 Jan 21;105(1):e70030. doi: 10.1111/cbdd.70030 (PMC11748363; doi:10.1111/cbdd.70030)
Supplement: Supplementary file 1 — Data S1. Biological assays—methods and full results, analytical data of prepared compounds, and representative NMR spectra in graphic form. [file CBDD-105-e70030-s001.pdf]

Antimicrobial and antiproliferative properties of 2-phenyl-*N*-(pyridin-2-yl)acetamides

Daria Nawrot[a], Barbora Koutníková[a], Ondřej Janďourek[a], Klára Konečná[a], Martin Novák[b], Pavla Paterová[c], Pavel Bárta[a], Ghada Bouz[a], Jan Zitko[a,*], Martin Doležal[a]

[a] Faculty of Pharmacy in Hradec Králové, Charles University, Heyrovského 1203, 500 05 Hradec Králové, Czech Republic

[b] Biomedical Research Centre, University Hospital Hradec Kralove, Sokolská 581, 500 05 Hradec Králové, Czech Republic

[c] Department of Clinical Microbiology, University Hospital Hradec Kralove, Sokolská 581, 500 05 Hradec Králové, Czech Republic

* Correspondence: jan.zitko@faf.cuni.cz (JZ)

SUPPLEMENTARY MATERIAL

Contents

1.1. Antimycobacterial *in vitro* activity – methodology ... 2
1.2. Antibacterial *in vitro* activity – methodology & full results ... 4
1.3. Antifungal *in vitro* activity – methodology & full results ... 6
1.4. Cytotoxicity determination ... 8
1.5. HPLC-MS stability determination in PBS (pH 7.4) ... 10
1.6. HPLC purity and HRMS determination ... 10
1.7. Analytical data of prepared compounds ... 11
1.8. ^{1}H NMR and ^{13}C NMR spectra of selected compounds ... 17
1.9. References ... 23

1.1. Antimycobacterial *in vitro* activity – methodology

Method for *M. smegmatis*, *M. aurum*, *M. tuberculosis* H37Ra

Antimycobacterial assay was performed with fast growing *Mycolicibacterium smegmatis* DSM 43465 (ATCC 607), *Mycolicibacterium aurum* DSM 43999 (ATCC 23366) from German Collection of Microorganisms and Cell Cultures (Braunschweig, Germany) and with avirulent strain of *Mycobacterium tuberculosis* H37Ra ITM-M006710 (ATCC 9431) from Belgian Co-ordinated Collections of Micro-organisms (Antwerpen, Belgium). The technique used for activity determination was microdilution broth panel method using 96-well microtitration plates based on EUCAST standards[1]. Culturing medium was Middlebrook 7H9 broth (Merck, Darmstadt, Germany) enriched with 0.4% of glycerol (Merck) and 10% of Middlebrook OADC growth supplement (Himedia, Mumbai, India).

Mycobacterial strains were cultured on Middlebrook 7H9 agar and suspensions were prepared in enriched Middlebrook 7H9 broth. Final density was adjusted to value 1.0 according to McFarland scale and diluted in ratio 1:20 (for fast growing mycobacteria) or 1:10 (for *M. tuberculosis*) with broth.

Tested compounds were dissolved in DMSO (Merck) then Middlebrook 7H9 broth was added to obtain concentration 2000 µg/mL. Standards used for activity determination were isoniazid (INH), rifampicin (RIF) and ciprofloxacin (CIP) (Merck). Final concentrations were reached by binary dilution and addition of mycobacterial suspension and were set as 500, 250, 125, 62.5, 31.25, 15.625, 7.81 and 3.91 µg/mL. Isoniazid was diluted in range 500-3.91 µg/mL for screening against fast growing mycobacteria and in range 1-0.0078 µg/mL for screening against *M. tuberculosis*. Rifampicin final concentrations ranged from 50 to 0.39 µg/mL for fast growing mycobacteria and from 1 to 0.0078 µg/mL for *M. tuberculosis*. Ciprofloxacin was used for screening antimycobacterial activity with the final concentrations 1, 0.5, 0.25, 0.125, 0.0625, 0.0313, 0.0156, 0.0078 µg/mL. The final concentration of DMSO did not exceed 2.5% (v/v) and did not affect the growth of *M. smegmatis*, *M. aurum* nor *M. tuberculosis*. Positive (broth, DMSO, bacteria) and negative (broth, DMSO) growth controls were included.

Plates containing mycobacterial strains were sealed with polyester adhesive film and all plates were incubated in dark at 37°C without agitation. The addition of 0.01% solution of resazurin sodium salt followed after 48 hours of incubation for *M. smegmatis*, after 72 hours of incubation for *M. aurum* and after 120 hours of incubation for *M. tuberculosis*, respectively. Stain was prepared by dissolving resazurin sodium salt (Merck) in deionized water to get 0.02% solution. Then 10% aqueous solution of Tween 80 (Merck) was prepared. Both liquids were mixed up making use of the same volumes and filtered through syringe membrane filter. Microtitration panels were then incubated for further 2.5 hours for determination of activity against M. smegmatis, 4 hours for *M. aurum* and 24 hours for M. tuberculosis, respectively.

Antimycobacterial activity was expressed as minimal inhibition concentration (MIC) and the value was read based on stain colour change (blue colour – active compound; pink colour – not active compound). All experiments were conducted in duplicate.

Method for *M. tuberculosis* H37Rv, *M. kansasii*, *M. avium*

Testing was performed according to the previously published method[2]. Tested strains *Mycobacterium tuberculosis* H37Rv CNCTC My 331/88 (ATCC 27294), *M. kansasii* CNCTC My 235/80 (ATCC 12478), *and M. avium ssp. avium* CNCTC My 80/72 (ATCC 15769) were obtained from the Czech National Collection of Type Cultures (CNCTC), National Institute of Public Health (Prague, Czech Republic). Middlebrook 7H9 broth of declared pH = 6.6 (Sigma-Aldrich) enriched with 0.4% of glycerol (Sigma-Aldrich) and 10% of OADC growth supplement (oleic acid, albumin, dextrose, catalase; Himedia, Mumbai, India) was used for cultivation. The tested compounds were dissolved and diluted in DMSO and mixed with broth (25 µL of DMSO solution in 4.475 mL of broth) and then placed (100 µL) into microplate wells. Mycobacterial inocula were suspended in isotonic saline solution and the density was adjusted to 0.5–1.0 according to the McFarland scale. These suspensions were diluted by 10-1 and used to inoculate the testing wells, by adding 100 µL of mycobacterial suspension per well. The final concentrations of tested compounds in wells were 100, 50, 25, 12.5, 6.25, 3.13, and 1.56 µg/mL. INH was used as a positive control (inhibition of growth). The negative control (visible growth) consisted of broth plus mycobacterial suspension plus DMSO (purity of broth). A total of 30 µL of Alamar Blue working solution (1:1 mixture of 0.01% resazurin sodium salt (aq. sol.) and 10% Tween 80) was added after five days of incubation. Results were determined after 24 h of incubation. The MIC (in µg/mL) was determined as the lowest concentration that prevented the blue-to-pink color change. The MIC values of INH were 6.25–12.5 µg/mL against *M. avium*, 3.13–12.5 µg/mL against *M. kansasii*, and 0.1–0.2 µg/mL against *M. tuberculosis* H37Rv. All experiments were conducted in duplicates.

1.2. Antibacterial *in vitro* activity – methodology & full results

Antibacterial assay was performed on: SA - *Staphylococcus aureus* CCM 4223, ATCC 29213, MRSA - *Staphylococcus aureus* subsp. aureus CCM 4750, ATCC 43300, SE - *Staphylococcus epidermidis* CCM 4418, ATCC 12228, EF - *Enterococcus faecalis* CCM 4224, ATCC 29212, EC - *Escherichia coli* CCM 3954, ATCC 25922, KP - *Klebsiella pneumoniae* CCM 4415, ATCC 10031, ACI - *Acinetobacter baumannii* DSM 30007, ATCC 19606, PA - *Pseudomonas aeruginosa* CCM 3955, ATCC 27853. Bacterial strains were purchased from the Czech Collection of Microorganisms (CCM) or the German Collection of Microorganisms and Cell Cultures (DSM, Braunschweig, Germany).

The microdilution broth method was performed according to EUCAST (The European Committee on Antimicrobial Susceptibility Testing) instructions[3], with slight modification. The cultivation was done in Cation-adjusted Mueller-Hinton broth (CAMHB, M-H 2 Broth, Sigma-Aldrich, USA) at 35±2°C. Tested compound and selected antibiotics (gentamicin – GEN, ciprofloxacin – CIP, Sigma-Aldrich, USA), used as internal quality controls were dissolved in DMSO (Sigma-Aldrich, USA) to produce stock solutions. The final concentration of DMSO in samples corresponded to 1% DMSO (*v*/*v*). Positive (microbes in CAMHB with final 1% DMSO, *v*/*v*), negative (CAMHB, 1% DMSO, *v*/*v*) controls, and internal quality standards were involved in assays. Antibacterial activity, expressed as minimum inhibitory concentration (MIC, reported in µM), was evaluated after 24 and 48 h of static incubation in the dark and humidified atmosphere at 35±2°C. Visual inspection and spectrophotometric measurement (530 nm, Synergy HTX Multi-Mode Microplate reader, BioTek, USA) were used for MIC endpoint evaluation.

Table S1. Results of internal quality controls (standards) in antibacterial screening

MIC (µg/mL)				
	Ciprofloxacin – spectrophotometric detection	Ciprofloxacin – visual detection	Gentamicin– spectrophotometric detection	Gentamicin – spectrophotometric detection
Staphylococcus aureus CCM 4223, ATCC 29213	0.256	0.256	1	0.5
Staphylococcus aureus subsp. aureus CCM 4750, ATCC 43300	0.128	0.128	>8	>8
Staphylococcus epidermidis CCM 4418, ATCC 12228	0.256	0.128	0.0625	0.0625
Enterococcus faecalis CCM 4224, ATCC 29212	1.024	1.024	>8	>8
Escherichia coli CCM 3954, ATCC 25922	0.008	0.008	1	1
Klebsiella pneumoniae CCM 4415, ATCC 10031	0.008	0.008	0.5	0.5
Acinetobacter baumannii DSM 30007, ATCC 19606	0.512	0.512	8	8
Pseudomonas aeruginosa CCM 3955, ATCC 27853	0.512	0.512	0.5	0.5

*The MIC of antibacterial agents is the lowest concentration giving rise to an inhibition of growth of 95% of that of the drug-free control. Results were read 24h after incubation without agitation at 35 ± 2 °C in a humidified atmosphere. Measured on a microplate reader (SynergyTM HTX, BioTek Instruments, Inc., USA) at wavelength 530 nm.

**The MIC was determined by the naked eye in the well with the lowest drug concentration, where no visible growth of microbial agent was detected. Results were read after 24h incubation without agitation at 35 ± 2 °C in a humidified atmosphere.

Table S2. Full results of the screening of antibacterial activity

	SA		MRSA		SE		EF		EC		KP		ACI		PA	
	µmol/L															
	24h	48h	24h	48h	24h	48h	24h	48h	24h	48h	24h	48h	24h	48h	24h	48h
1	>500	>500	>500	>500	>500	>500	>500	>500	>500	>500	>500	>500	>500	>500	>500	>500
2	>500	>500	>500	>500	>500	>500	>500	>500	>500	>500	>500	>500	>500	>500	>500	>500
3	**500**	**500**	>500	>500	>500	>500	>500	>500	>500	>500	>500	>500	>500	>500	>500	>500
4	>500	>500	>500	>500	>500	>500	>500	>500	>500	>500	>500	>500	>500	>500	>500	>500
5	>500	>500	>500	>500	>500	>500	>500	>500	>500	>500	>500	>500	>500	>500	>500	>500
6	>500	>500	>500	>500	>500	>500	>500	>500	>500	>500	>500	>500	>500	>500	>500	>500
7	**125**	**125**	>125	>125	>125	>125	>125	>125	>125	>125	>125	>125	>125	>125	>125	>125
8	**500**	>500	>500	>500	**250**	>500	>500	>500	>500	>500	>500	>500	>500	>500	>500	>500
9	>500	>500	>500	>500	>500	>500	>500	>500	>500	>500	>500	>500	>500	>500	>500	>500
10	>500	>500	>500	>500	**500**	**500**	>500	>500	>500	>500	>500	>500	>500	>500	>500	>500
11	**250**	**500**	>500	>500	>500	>500	>500	>500	>500	>500	>500	>500	>500	>500	>500	>500
12	**500**	**500**	**500**	>500	**500**	>500	**500**	**500**	>500	>500	>500	>500	>500	>500	>500	>500
13	>500	>500	>500	>500	**500**	**500**	>500	>500	>500	>500	>500	>500	>500	>500	>500	>500
14	>500	>500	>500	>500	>500	>500	>500	>500	>500	>500	>500	>500	>500	>500	>500	>500
15	>500	>500	>500	>500	>500	>500	>500	>500	>500	>500	>500	>500	>500	>500	>500	>500
16	500	500	>500	>500	**125**	**250**	>500	>500	>500	>500	>500	>500	>500	>500	>500	>500
17	>500	>500	>500	>500	>500	>500	>500	>500	>500	>500	>500	>500	>500	>500	>500	>500
18	>500	>500	>500	>500	>500	>500	>500	>500	>500	>500	>500	>500	>500	>500	>500	>500
19	**500**	**500**	>500	>500	**125**	**125**	>500	>500	>500	>500	>500	>500	>500	>500	>500	>500
20	>500	>500	>500	>500	>500	>500	>500	>500	>500	>500	>500	>500	>500	>500	>500	>500
21	**250**	>500	**250**	**500**	>500	>500	>500	>500	>500	>500	>500	>500	>500	>500	>500	>500
22	>500	>500	>500	>500	>500	>500	>500	>500	>500	>500	>500	>500	>500	>500	>500	>500
23	>500	>500	>500	>500	>500	>500	>500	>500	>500	>500	>500	>500	>500	>500	>500	>500
24	>500	>500	>500	>500	>500	>500	>500	>500	>500	>500	>500	>500	>500	>500	>500	>500
25	>500	>500	>500	>500	>500	>500	>500	>500	>500	>500	>500	>500	>500	>500	>500	>500
26	>500	>500	>500	>500	>500	>500	>500	>500	>500	>500	>500	>500	>500	>500	>500	>500
27	>500	>500	>500	>500	**250**	**250**	>500	>500	>500	>500	>500	>500	>500	>500	>500	>500
28	>500	>500	>500	>500	>500	>500	>500	>500	>500	>500	>500	>500	>500	>500	>500	>500
29	>500	>500	>500	>500	**250**	**250**	>500	>500	>500	>500	>500	>500	>500	>500	>500	>500
30	>500	>500	>500	>500	>500	>500	>500	>500	>500	>500	>500	>500	>500	>500	>500	>500
31	**500**	>500	>500	>500	**250**	>500	>500	>500	>500	>500	>500	>500	>500	>500	>500	>500
32	>500	>500	>500	>500	**125**	**125**	>500	>500	>500	>500	>500	>500	>500	>500	>500	>500
33	>500	>500	>500	>500	>500	>500	>500	>500	>500	>500	>500	>500	>500	>500	>500	>500

SA - *Staphylococcus aureus*, MRSA - methicillin-resistant *Staphylococcus aureus*, SE - *Staphylococcus epidermidis,* EF - *Enterococcus faecalis,* EC -*Escherichia coli*, KP - *Klebsiella pneumonia* ACI - *Acinetobacter baumannii*, PA - *Pseudomonas aeruginosa*.

1.3. Antifungal *in vitro* activity – methodology & full results

Antifungal assay was performed on: CA - *Candida albicans* CCM 8320, ATCC 24433; CK - *Candida krusei* CCM 8271, ATCC 6258; CP - *Candida parapsilosis* CCM 8260, ATCC 22019; CT - *Candida tropicalis* CCM 8264, ATCC 750; AF - Aspergillus *fumigatus* ATCC 204305; Afla - *Aspergillus flavus* CCM 8363, LC - *Lichtheimia corymbifera* CCM 8077; TI - *Trichophyton interdigitale* CCM 8377, ATCC 9533. Fungal strains were obtained from the Czech Collection of Microorganisms (CCM, Brno, Czech Republic) and American Type Culture Collection (ATCC, Mannasas, VA, USA). The antifungal activity was evaluated by the microdilution broth method according to EUCAST instructions[4,5] with slight modification. Tested compounds were dissolved in DMSO and diluted in a twofold manner with RPMI (Roswell Park Memorial Institute) 1640 medium, with glutamine and 2% glucose, buffered to pH 7.0 with MOPS (3-morpholinopropane-1-sulfonic acid). The final concentration of DMSO in the testing medium did not exceed 1% (v/v) of the total solution composition. Static incubation was performed in the dark, and in humid atmosphere, at 35±2 °C, for 24 and 48 h (72 and 120 h for *Trichophyton interdigitale* respectively). Positive controls consisted of test microbe solely, while negative controls consisted of cultivation medium and DMSO. Visual inspection was used to evaluate MIC. The internal quality standards, amphotericin B (AMB) and voriconazole (VRC) were used in the assays.

Table S3. Results of internal quality controls (standards) in antifungal screening.

	MIC (μg/mL)			
	Amphotericin B - IC_{90} spectrophotometric detection*	Amphotericin B - visual detection**	Voriconazole - IC_{90} spectrophotometric detection***	Voriconazole - visual detection**
Candida albicans CCM 8320, ATCC 24433	1	1	0.03	>16
Candida krusei CCM 8271, ATCC 6258	1	1	0.25	>16
Candida parapsilosis CCM 8260, ATCC 22019	0.5	0.5	0.03	>16
Candida tropicalis CCM 8264, ATCC 750	1	1	2	>16
Aspergillus *fumigatus* ATCC 204305	4	4	0.25	1
Aspergillus flavus CCM 8363	4	4	2	>16
Lichtheimia corymbifera CCM 8077	1	1	>16	>16
Trichophyton interdigitale CCM 8377, ATCC 9533	1	1	1	>16

*The IC_{90} of amphotericin B was determined as the lowest concentration giving rise to an inhibition of growth of 90% of that of the drug-free control. Results were read after 24h (yeasts) or 48h (moulds) without agitation at 35 ± 2 °C in a humidified atmosphere. Measured on a microplate reader (SynergyTM HTX, BioTek Instruments, Inc., USA) at wavelength 530 nm.

**The MIC was determined by the naked eye in the well with the lowest drug concentration, where no visible growth of microbial agent was detected. Results were read after 24h (yeasts) or 48h (moulds) of incubation without agitation at 35 ± 2 °C in a humidified atmosphere.

***The IC_{50} of voriconazole was determined as the lowest drug concentration giving rise to an inhibition of growth of 50% compared to the drug-free control. Results were read after 24h (yeasts) or 48h (moulds) cultivation without agitation at 35±2°C in a humidified atmosphere. Measured on amicroplate reader (SynergyTM HTX, BioTek Instruments, Inc., USA) at wavelength 530 nm.

Table S4. Full results of the screening of antifungal activity

	CA		CK		CP		CT		AF		AFla		LC		TI	
	μmol/L															
	24h	48h	24h	48h	24h	48h	24h	48h	24h	48h	24h	48h	24h	48h	72h	120h
1	**500**	**500**	**500**	**500**	**500**	**500**	**500**	**500**	>500	>500	>500	>500	>500	>500	**500**	**500**
2	>500	>500	>500	>500	>500	>500	>500	>500	>500	>500	>500	>500	>500	>500	>500	>500
3	>500	>500	**250**	**500**	**500**	>500	>500	>500	>500	>500	>500	>500	>500	>500	125	250
4	>500	>500	>500	>500	>500	>500	>500	>500	>500	>500	>500	>500	>500	>500	>500	>500
5	>500	>500	>500	>500	>500	>500	>500	>500	>500	>500	>500	>500	>500	>500	**500**	**500**
6	>500	>500	>500	>500	>500	>500	>500	>500	>500	>500	>500	>500	>500	>500	>500	>500
7	>125	>125	>125	>125	>125	>125	>125	>125	>125	>125	>125	>125	>125	>125	>125	>125
8	>500	>500	**500**	**500**	>500	>500	>500	>500	>500	>500	**500**	**500**	**500**	**500**	**500**	**500**
9	**500**	**500**	**500**	**500**	**500**	**500**	**500**	**500**	**500**	>500	**500**	>500	**500**	>500	**500**	>500
10	>500	>500	**500**	**500**	>500	>500	>500	>500	>500	>500	>500	>500	>500	>500	**500**	**500**
11	>500	>500	>500	>500	>500	>500	>500	>500	>500	>500	>500	>500	>500	>500	>500	>500
12	>500	>500	>500	>500	>500	>500	>500	>500	>500	>500	>500	>500	>500	>500	>500	>500
13	>500	>500	>500	>500	>500	>500	>500	>500	>500	>500	>500	>500	>500	>500	500	500
14	>500	>500	>500	>500	>500	>500	>500	>500	>500	>500	>500	>500	>500	>500	>500	>500
15	>500	>500	500	500	>500	>500	>500	>500	>500	>500	>500	>500	>500	>500	**500**	**500**
16	>500	>500	>500	>500	>500	>500	>500	>500	>500	>500	>500	>500	>500	>500	**500**	**500**
17	>500	>500	>500	>500	>500	>500	>500	>500	>500	>500	>500	>500	>500	>500	**500**	**500**
18	>500	>500	>500	>500	>500	>500	>500	>500	>500	>500	>500	>500	>500	>500	**500**	**500**
19	>500	>500	**500**	**500**	**500**	**500**	>500	>500	>500	>500	>500	>500	>500	>500	**250**	**250**
20	>500	>500	>500	>500	>500	>500	>500	>500	>500	>500	>500	>500	>500	>500	>500	>500
21	>500	>500	>500	>500	>500	>500	>500	>500	>500	>500	>500	>500	>500	>500	**62.5**	**125**
22	>500	>500	>500	>500	>500	>500	>500	>500	500	>500	>500	>500	500	>500	**500**	**500**
23	>500	>500	>500	>500	>500	>500	>500	>500	>500	>500	>500	>500	>500	>500	**500**	**500**
24	>500	>500	>500	>500	>500	>500	>500	>500	>500	>500	>500	>500	>500	>500	**500**	**500**
25	>500	>500	>500	>500	>500	>500	>500	>500	>500	>500	>500	>500	>500	>500	**500**	**500**
26	>500	>500	>500	>500	>500	>500	>500	>500	>500	>500	>500	>500	>500	>500	**500**	**500**
27	>500	>500	>500	>500	>500	>500	>500	>500	>500	>500	>500	>500	>500	>500	>500	>500
28	>500	>500	>500	>500	>500	>500	>500	>500	>500	>500	>500	>500	>500	>500	>500	>500
29	>500	>500	>500	>500	>500	>500	>500	>500	>500	>500	>500	>500	>500	>500	>500	>500
30	>500	>500	>500	>500	>500	>500	>500	>500	>500	>500	>500	>500	>500	>500	**500**	**500**
31	>500	>500	**500**	**500**	**500**	>500	>500	>500	>500	>500	>500	>500	>500	>500	>500	>500
32	>500	>500	>500	>500	>500	>500	>500	>500	>500	>500	>500	>500	>500	>500	>500	>500
33	>500	>500	**31.25**	**62.5**	>500	>500	>500	>500	>500	>500	>500	>500	>500	>500	>500	>500

CA - *Candida albicans*, CK - *Candida krusei*, CP - *Candida parapsilosis*, CT - *Candida tropicalis*, AF - Aspergillus *fumigatus,* Afla - *Aspergillus flavus*, LC - *Lichtheimia corymbifera,* TI - *Trichophyton interdigitale*.

1.4. Cytotoxicity determination

HepG2

Human hepatocellular liver carcinoma cell line HepG2 purchased from Health Protection Agency Culture Collections (ECACC, Salisbury, UK) was cultured in MEM (Minimum Essentials Eagle Medium, Sigma–Aldrich) supplemented with 10% fetal bovine serum (PAA Laboratories, Pasching, Austria), 1% L-glutamine solution (Sigma–Aldrich) and non-essential amino acid solution (Sigma–Aldrich) in a humidified atmosphere containing 5% CO_2 at 37 °C. For subculturing, the cells were harvested after trypsin/EDTA (Sigma–Aldrich) treatment at 37 °C. To evaluate cytotoxicity, the cells treated with the tested substances were used as experimental groups whereas untreated HepG2 cells served as controls. The cells were seeded in density 10,000 cells per well in a 96 well plate. The next day the cells were treated with each of the tested substances dissolved in DMSO. The tested substances were prepared at different incubation concentrations (0.0001, 1, 5, 10, 25, 50, 100, 250, 500 and 1000 µM) in triplicates according to their solubility. Simultaneously, the controls representing 100% cell viability, 0% cell viability (the cells treated with 10% DMSO), no cell control and vehiculum controls were also prepared in triplicates. After 24 h incubation in a humidified atmosphere containing 5% CO_2 at 37%, the reagent from the kit CellTiter 96 AQueous One Solution Cell Proliferation Assay (CellTiter 96; PROMEGA, Fitchburg, USA) was added. After 2h incubation at 37 °C, absorbance of samples was recorded at 490 nm (TECAN, Infinita M200, Austria). A standard toxicological parameter IC_{50} was calculated by nonlinear regression from a semilogarithmic plot of incubation concentration versus percentage of absorbance relative to untreated controls using GraphPad Prism 8 software.

Other cell lines

Human epithelial kidney carcinoma A498 cell line, obtained from American Type Culture Collection (ATCC, Manassas, VA, USA, catalogue No.: HTB-44), was cultured in RPMI medium, Human normal kidney proximal tubule HK-2 cell line obtained from ATCC (ATCC, Manassas, VA, USA, catalogue No.: CRL-2190) was cultured in DMEM High Glucose medium, Human Caucasian prostate adenocarcinoma PC-3 cell line, obtained from ATCC (Manassas, VA, USA, catalogue No.: 90112714), was cultured in a Kaighn's Modification of Ham's F-12 Medium and human glioblastoma astrocytoma U-87 MG cell line, obtained from ECACC (ECACC, Salisbury, UK, catalogue No.: 89081402) was cultured in DMEM High Glucose medium. All cultures were supplemented with 10% fetal bovine serum, 1% non-essential amino acids (A498, HK-2 and U-87 MG), 1% sodium pyruvate (A498, HK-2 and U-87 MG) and 2 mM L-glutamine. All cell culture consumables were purchased from Merck (Darmstadt, Germany). The cells were kept at 37 °C in a 5% CO2 humidified incubator and were subcultured and used for experiments at a confluency of 70–90%. The cells were seeded in density 10,000 cells per well in a 96 well plate in the cell culture medium and left to incubate for 24 hours. The tested compound was added in the chosen concentrations (0.0001, 1, 5, 10, 25, 50, 100, 250, 500 and 1000 µM in triplicates). Simultaneously, the controls representing 100% cell viability, 0% cell viability (the cells treated with 10% DMSO), no cell control and vehiculum controls were also prepared in triplicates. Cells were incubated for the next 24 hours. Eventually, a colorimetric assay reagent CellTiter 96 AQueous One Solution Cell Proliferation Assay was added, and absorption was measured.

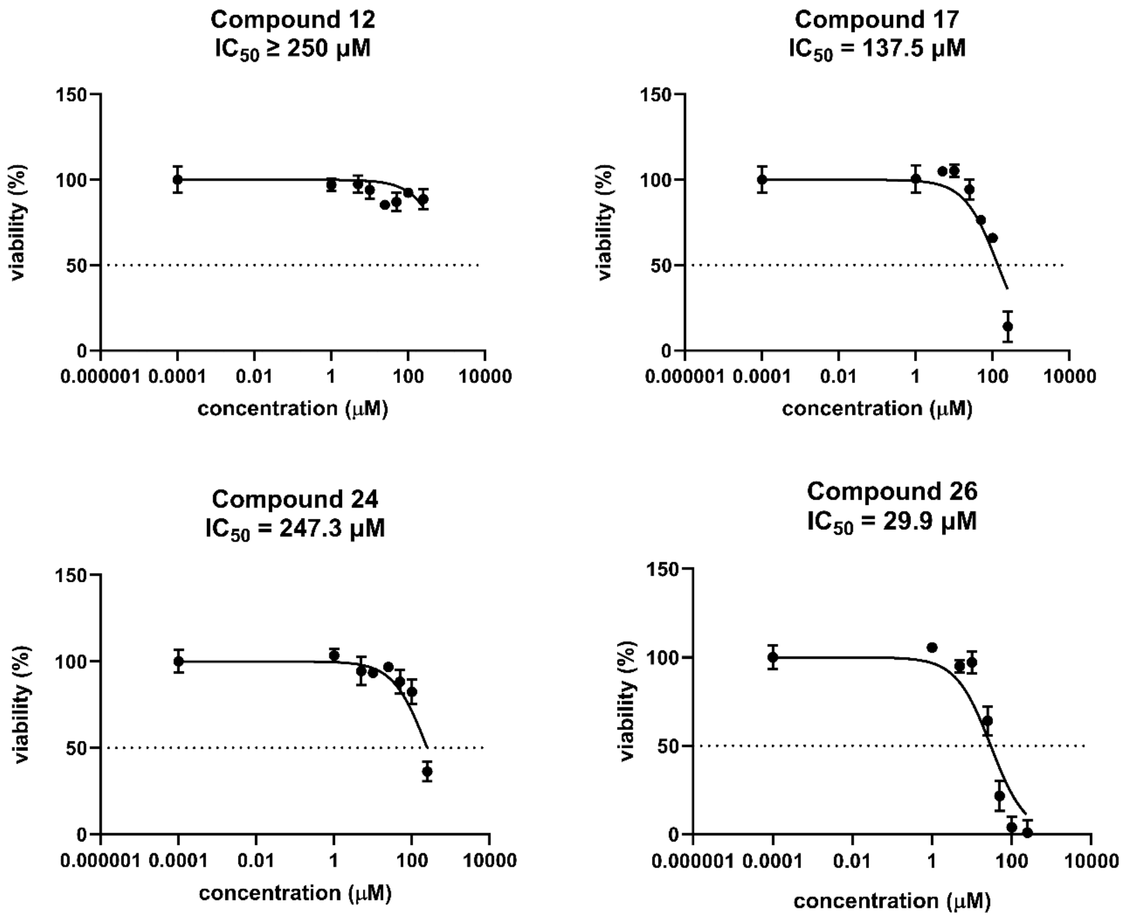

Figure S1. Viability curves for compounds **12**, **17**, **24**, and **26** in HK-2 cel line

1.5. HPLC-MS stability determination in PBS (pH 7.4)

Compounds **12** and **24** were incubated in phosphate-buffered saline (PBS) pH 7.4 to determine their stability. Briefly, compounds were dissolved in DMSO to produce stock sample solutions. 10 µL of stock solution was added to 1990 µL PBS solution. pH of PBS solution was adjusted to 7.4 by addition of NaOH. The concentration of tested compounds was set at 1 µM. Each compound was incubated for 5, 15, 30, 60, 90, 120 and 180 min at 37 °C. The reactions were stopped by transferring 100 µL of incubate to 200 µL of acetonitrile containing internal standard (IS; compound 67 from ref[1]) at the appropriate time points and centrifuged at 12,000 rpm for 10 min at 4 °C. After that, 150 µL of supernatant was transferred to the vial and analyzed by LC-MS. The areas of the compounds (A_{cmpd}) and internal standards (A_{IS}) were detected in extracted ion chromatograms from the mass spectrometer data in positive mode.

LC-MS analysis: The samples were analyzed by an LC-MS system consisting of UHLPC Dionex Ultimate 3000 RS coupled with a Q Exactive Plus orbitrap mass spectrometer to obtain the areas (Thermo Fisher Scientific, Bremen, Germany). Reverse-phase C18 column Kinetex EVO (Phenomenex, Torrance, CA, USA) was used as a stationary phase, and purified water with 0.1% formic acid (mobile phase A) and LC-MS grade acetonitrile with 0.1% formic acid (mobile phase B) were used as the mobile phases. Gradient elution was used to determine purities and mass spectra. The method started with 5% B for 0.3 min, then the gradient switched to 100% B in the third min, remained at 100% B for 0.7 min and then went back to 5% B with equilibration for 3.5 min. The total run time of the method was 7.5 min. The column temperature was kept constant at 27 °C, the flow of the mobile phase was 0.5 mL/min, and the injection volume was 1 µL. HRMS spectra were collected from the total ion current in the scan range 105–1000 m/z, with the resolution set to 140,000.

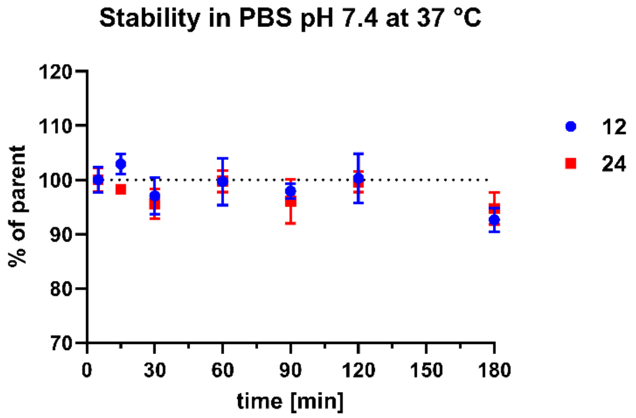

Figure S2. Stability of compounds **12** (blue) and **24** (red) in phosphate-buffered saline pH 7.4 at 37 °C

1.6. HPLC purity and HRMS determination

The purity and the experimental m/z values of synthesized compounds were determined using the same LC-MS system and the analytical method mentioned above.

1.7. Analytical data of prepared compounds

2-phenyl-*N*-(pyridin-2-yl)acetamide (**1**)

mp. 120.4–122.7 °C; Yield 88%; ^{1}H NMR (500 MHz, $CDCl_3$) δ 8.26–8.20 (m, 2H, ArH), 8.17 (s, 1H, CONH), 7.73–7.66 (m, 1H, ArH), 7.43–7.36 (m, 2H, ArH), 7.36–7.29 (m, 3H, ArH), 7.05–6.99 (m, 1H, ArH), 3.76 (s, 2H, CH_2); ^{13}C NMR (126 MHz, $CDCl_3$) δ 169.50, 151.22, 147.67, 138.35, 133.92, 129.41, 129.17, 127.65, 119.86, 114.00, 44.00; IR (ATR-Ge, cm^{-1}): 3242 (ν, NH), 1667 (ν, C=O, amide), 1609, 1585, 1544; Calculated for $C_{13}H_{12}N_2O$ (MW 212.23): 73.57% C; 5.69% H; 13.20% N; found 73.21% C; 5.38% H; 13.14% N; CAS Registry Number 5223-01-8.

2-phenyl-*N*-(5-chloropyridin-2-yl)acetamide (**2**)

mp. 121.0–123.0 °C; Yield 69%; ^{1}H NMR (500 MHz, $CDCl_3$) δ 8.22 (d, *J* = 8.9 Hz, 1H, ArH), 8.16 (d, *J* = 2.6 Hz, 1H, ArH), 7.96 (s, 1H, CONH), 7.68–7.62 (m, 1H, ArH), 7.44–7.37 (m, 2H, ArH), 7.37–7.30 (m, 3H, ArH), 3.77 (s, 2H, CH_2); ^{13}C NMR (126 MHz, $CDCl_3$) δ 169.40, 149.43, 146.31, 137.95, 133.64, 129.42, 129.26, 127.81, 126.84, 114.56, 44.86; IR (ATR-Ge, cm^{-1}): 3252 (ν, NH), 1699 (ν, C=O, amide), 1592, 1576, 1522; Calculated for $C_{13}H_{11}ClN_2O$ (MW 246.68): 63.29% C; 4.49% H; 11.36% N; found 63.50% C; 4.36% H; 11.21% N; CAS Registry Number 258338-50-0.

2-phenyl-*N*-(6-chloropyridin-2-yl)acetamide (**3**)

mp. 85.0–88.1 °C; Yield 42%; ^{1}H NMR (500 MHz, $CDCl_3$) δ 8.17 (d, *J* = 8.2 Hz, 1H, ArH), 7.94 (s, 1H, CONH), 7.65 (t, *J* = 8.0 Hz, 1H, ArH), 7.43–7.34 (m, 2H, ArH), 7.37–7.31 (m, 2H, ArH), 7.34–7.26 (m, 1H, ArH), 7.07–7.02 (m, 1H, ArH), 3.76 (s, 2H, CH_2); ^{13}C NMR (126 MHz, $CDCl_3$) δ 169.55, 150.99, 148.81, 140.89, 133.47, 129.40, 129.24, 127.81, 119.80, 111.98, 44.92; IR (ATR-Ge, cm^{-1}): 3231 (ν, NH), 1661 (ν, C=O, amide), 1571, 1538, 1495; Calculated for $C_{13}H_{11}ClN_2O$ (MW 246.68): 63.29% C; 4.49% H; 11.36% N; found 63.35% C; 4.78% H; 11.34% N; CAS Registry Number 1710904-24-7.

2-(2-methoxyphenyl)-*N*-(pyridin-2-yl)acetamide (**4**)

mp. 91.3–93.7 °C; Yield 52%; ^{1}H NMR (600 MHz, DMSO) δ 10.40 (s, 1H, CONH), 8.29–8.25 (m, 1H, ArH), 8.00 (d, J = 8.4 Hz, 1H, ArH), 7.74–7.67 (m, 1H, ArH), 7.24–7.14 (m, 2H, ArH), 7.07–7.01 (m, 1H, ArH), 6.94 (d, J = 7.1 Hz, 1H, ArH), 6.90–6.82 (m, 1H, ArH), 3.71 (s, 3H, OCH_3), 3.68 (s, 2H, CH_2); ^{13}C NMR (151 MHz, DMSO) δ 170.54, 157.84, 152.67, 148.48, 138.64, 131.52, 128.66, 124.53, 120.73, 119.74, 113.82, 111.29, 55.97, 38.20; IR (ATR-Ge, cm^{-1}): 3041 (ν, NH), 1714 (ν, C=O, amide), 1598, 1583, 1559; Calculated for $C_{14}H_{14}N_2O_2$ (MW 242.26): 69.41% C; 5.82% H; 11.57% N; found 69.62% C; 5.64% H; 11.38% N; CAS Registry Number 346700-10-5.

2-(2-methoxyphenyl)-*N*-(5-chloropyridin-2-yl)acetamide (**5**)

mp. 90.6–91.3 °C; Yield 28%; ^{1}H NMR (600 MHz, DMSO) δ 10.64 (s, 1H, CONH), 8.34–8.30 (m, 1H, ArH), 8.04 (d, *J* = 8.9 Hz, 1H, ArH), 7.86–7.79 (m, 1H, ArH), 7.24–7.13 (m, 2H, ArH), 6.96–6.90 (m, 1H, ArH), 6.90–6.81 (m, 1H, ArH), 3.71 (s, 3H, OCH_3), 3.68 (s, 2H, CH_2); ^{13}C NMR (151 MHz, DMSO) δ 170.71, 157.84, 151.34, 146.84, 138.39, 131.54, 128.71, 125.37, 124.36, 120.72, 114.91, 111.29, 55.97, 38.14; IR (ATR-Ge, cm^{-1}): 3305 (ν, NH), 1702 (ν, C=O, amide), 1576, 1524, 1517; Calculated for $C_{14}H_{13}ClN_2O_2$ (MW 276.72): 60.55% C; 5.08% H; 10.09% N; found 60.38% C; 4.73% H; 9.95% N; CAS Registry Number 349124-92-1.

2-(2-methoxyphenyl)-*N*-(6-chloropyridin-2-yl)acetamide (**6**)

mp. 110.1–112.5 °C; Yield 92%; ^{1}H NMR (500 MHz, DMSO) δ 10.87 (s, 1H, CONH), 8.02 (d, *J* = 8.2 Hz, 1H, ArH), 7.81 (t, *J* = 8.0 Hz, 1H, ArH), 7.27–7.21 (m, 1H, ArH), 7.21–7.15 (m, 2H, ArH), 6.96 (d, *J* = 8.0 Hz, 1H, ArH), 6.92–6.85 (m, 1H, ArH), 3.73 (s, 3H, OCH_3), 3.70 (s, 2H, CH_2); ^{13}C NMR (126 MHz, DMSO)

δ 170.78, 157.76, 152.65, 148.42, 142.16, 131.50, 128.64, 124.19, 120.61, 119.31, 112.28, 111.16, 55.88, 38.02; IR (ATR-Ge, cm^{-1}): 3280 (ν, NH),1669 (ν, C=O, amide), 1590, 1572, 1526; Calculated for $C_{14}H_{13}ClN_2O_2$ (MW 276.72): 60.55% C; 5.08% H; 10.09% N; found 60.59% C; 4.70% H; 10.16% N; CAS Registry Number 1713531-95-3.

2-(3-methoxyphenyl)-*N*-(pyridin-2-yl)acetamide (**7**)

mp. 91.0–93.0 °C; Yield 50%; ^{1}H NMR (500 MHz, $CDCl_3$) δ 8.26–8.19 (m, 2H, ArH), 8.17 (s, 1H, CONH), 7.73–7.66 (m, 1H, ArH), 7.34–7.26 (m, 1H, ArH), 7.05–7.00 (m, 1H, ArH), 6.93–6.89 (m, 1H, ArH), 6.88–6.84 (m, 2H, ArH), 3.81 (s, 3H, OCH_3), 3.73 (s, 2H, CH_2); ^{13}C NMR (126 MHz, $CDCl_3$) δ 169.38, 160.11, 151.21, 147.63, 138.35, 135.32, 130.23, 121.65, 119.85, 115.07, 113.98, 113.17, 55.19, 44.97; IR (ATR-Ge, cm^{-1}): 3239 (ν, NH), 1658 (ν, C=O, amide), 1598, 1576, 1539; Calculated for $C_{14}H_{14}N_2O_2$ (MW 242.26): 69.41% C; 5.82% H; 11.57% N; found 69.19% C; 5.54% H; 11.21% N; CAS Registry Number 1060911-91-2.

2-(3-methoxyphenyl)-*N*-(5-chloropyridin-2-yl)acetamide (**8**)

mp. 103.0–104.0 °C; Yield 37%; ^{1}H NMR (500 MHz, $CDCl_3$) 8.22 (d, J = 8.9 Hz, 1H, ArH), 8.16 (d, J = 2.6 Hz, 1H, ArH), 7.93 (s, 1H, CONH), 7.68–7.62 (m, 1H, ArH), 7.32 (t, J = 7.8 Hz, 1H, ArH), 6.94–6.84 (m, 3H, ArH), 3.82 (s, 3H, OCH_3), 3.74 (s, 2H, CH_2); ^{13}C NMR (126 MHz, $CDCl_3$) δ 169.27, 160.20, 149.42, 146.32, 137.93, 135.04, 130.36, 126.84, 121.65, 115.16, 114.54, 113.26, 55.23, 44.94; IR (ATR-Ge, cm^{-1}): 3257 (ν, NH), 1698 (ν, C=O, amide), 1598, 1579, 1524; Calculated for $C_{14}H_{13}ClN_2O_2$ (MW 276.72): 60.55% C; 5.08% H; 10.09% N; found 60.71% C; 4.69% H; 9.99% N; CAS Registry Number 1623375-59-6.

2-(3-methoxyphenyl)-*N*-(6-chloropyridin-2-yl)acetamide (**9**)

mp. 70.0–72.0 °C; Yield 22%; ^{1}H NMR (500 MHz, $CDCl_3$) δ 8.17 (d, J = 8.2 Hz, 1H, ArH), 7.90 (s, 1H, CONH), 7.65 (t, J = 8.0 Hz, 1H, ArH), 7.31 (t, J = 7.9 Hz, 1H, ArH), 7.04 (d, J = 7.7 Hz, 1H, ArH), 6.94–6.83 (m, 3H, ArH), 3.83 (s, 3H, OCH_3), 3.73 (s, 2H, CH_2); ^{13}C NMR (126 MHz, $CDCl_3$) δ 169.42, 160.17, 150.97, 148.82, 140.88, 134.83, 130.34, 121.65, 119.81, 115.14, 113.28, 111.97, 55.23, 45.01; IR (ATR-Ge, cm^{-1}): 3232 (ν, NH), 1662 (ν, C=O, amide), 1594, 1572, 1437; Calculated for $C_{14}H_{13}ClN_2O_2$ (MW 276.72): 60.55% C; 5.08% H; 10.09% N; found 60.37% C; 4.68% H; 9.78% N; CAS Registry Number 1710438-80-4.

2-(4-methoxyphenyl)-*N*-(pyridin-2-yl)acetamide (**10**)

mp. 83.0–84.0 °C; Yield 44%; ^{1}H NMR (500 MHz, $CDCl_3$) δ 8.26–8.19 (m, 2H, ArH), 8.16 (s, 1H, CONH), 7.73–7.66 (m, 1H, ArH), 7.35–7.20 (m, 2H, ArH), 7.10–6.99 (m, 1H, ArH), 6.95–6.88 (m, 2H, ArH), 3.82 (s, 3H, OCH_3), 3.70 (s, 2H, CH_2); ^{13}C NMR (126 MHz, $CDCl_3$) δ 169.98, 159.09, 151.23, 147.56, 138.41, 130.56, 125.86, 119.80, 114.62, 113.95, 55.26, 44.01; IR (ATR-Ge, cm^{-1}): 3231 (ν, NH), 1659 (ν, C=O, amide), 1578, 1540, 1514; Calculated for $C_{14}H_{14}N_2O_2$ (MW 242.26): 69.41% C; 5.82% H; 11.57% N; found 69.28% C; 5.51% H; 11.42% N; CAS Registry Number 305358-98-9.

2-(4-methoxyphenyl)-*N*-(5-chloropyridin-2-yl)acetamide (**11**)

mp. 139.0–140.0 °C; Yield 59%; ^{1}H NMR (500 MHz, $CDCl_3$) δ 8.22 (d, J = 8.9 Hz, 1H, ArH), 8.17–8.13 (m, 1H, ArH), 7.93 (s, 1H, CONH), 7.68–7.62 (m, 1H, ArH), 7.29–7.20 (m, 2H, ArH), 6.96–6.89 (m, 2H, ArH), 3.82 (s, 3H, OCH_3), 3.70 (s, 2H, CH_2); ^{13}C NMR (126 MHz, $CDCl_3$) δ 169.87, 159.20, 149.46, 146.30, 137.93, 130.58, 126.79, 125.53, 114.72, 114.52, 55.27, 43.98; IR (ATR-Ge, cm^{-1}): 3252 (ν, NH), 1696 (ν, C=O, amide), 1579, 1528, 1513; Calculated for $C_{14}H_{13}ClN_2O_2$ (MW 276.72): 60.55% C; 5.08% H; 10.09% N; found 60.83% C; 5.13% H; 9.88% N; CAS Registry Number 349124-66-9.

2-(4-methoxyphenyl)-*N*-(6-chloropyridin-2-yl)acetamide (**12**)

mp. 84.0–85.0 °C; Yield 20%; ^{1}H NMR (500 MHz, $CDCl_3$) δ 8.17 (d, *J* = 8.2 Hz, 1H, ArH), 7.92 (s, 1H, CONH), 7.64 (t, *J* = 8.0 Hz, 1H, ArH), 7.28–7.19 (m, 2H, ArH), 7.07–7.01 (m, 1H, ArH), 6.95–6.88 (m, 2H, ArH), 3.83 (s, 3H, OCH_3), 3.70 (s, 2H, CH_2); ^{13}C NMR (126 MHz, $CDCl_3$) δ 170.01, 159.20, 151.02, 148.79, 140.87, 130.55, 125.37, 119.75, 114.70, 111.94, 55.28, 44.04; IR (ATR-Ge, cm^{-1}): 3232 (ν, NH), 1612 (ν, C=O, amide), 1572, 1540, 1514; Calculated for $C_{14}H_{13}ClN_2O_2$ (MW 276.72): 60.55% C; 5.08% H; 10.09% N; found 60.91% C; 4.69% H; 9.96% N; HPLC purity: 98.09%; HRMS (ESI$^+$): $[M+H]^+$ calcd. for $C_{14}H_{14}ClN_2O_2^+$ (m/z): 277.07383, found 277.07672; CAS Registry Number 1713741-77-5.

2-(2-chlorophenyl)-*N*-(pyridin-2-yl)acetamide (**13**)

mp. 97.6–100.6 °C; Yield 50%; ^{1}H NMR (600 MHz, DMSO) δ 10.68 (s, 1H, CONH), 8.33–8.25 (m, 1H, ArH), 8.00 (d, *J* = 8.4 Hz, 1H, ArH), 7.75–7.68 (m, 1H, ArH), 7.43–7.32 (m, 2H, ArH), 7.31–7.21 (m, 2H, ArH), 7.11–7.01 (m, 1H, ArH), 3.89 (s, 2H, CH_2); ^{13}C NMR (151 MHz, DMSO) δ 169.40, 152.57, 148.53, 138.71, 134.34, 134.29, 132.83, 129.55, 129.17, 127.60, 119.91, 113.89, 41.19; IR (ATR-Ge, cm^{-1}): 3261 (ν, NH), 1696 (ν, C=O, amide), 1615, 1596, 1580; Calculated for $C_{13}H_{11}ClN_2O$ (MW 246.68): 63.29% C; 4.49% H; 11.36% N; found 63.25% C; 4.38% H; 10.99% N; CAS Registry Number 775301-69-4.

2-(2-chlorophenyl)-*N*-(5-chloropyridin-2-yl)acetamide (**14**)

mp. 124.1–125.9 °C; Yield 12%; ^{1}H NMR (600 MHz, DMSO) δ 10.89 (s, 1H, CONH), 8.34 (d, *J* = 2.6 Hz, 1H, ArH), 8.03 (d, *J* = 8.9 Hz, 1H, ArH), 7.88–7.81 (m, 1H, ArH), 7.43–7.34 (m, 2H, ArH), 7.31–7.22 (m, 2H, ArH), 3.90 (s, 2H, CH_2);^{13}C NMR (151 MHz, DMSO) δ 169.56, 151.21, 146.90, 138.47, 134.28, 134.14, 132.85, 129.55, 129.23, 127.62, 125.57, 114.98, 41.14; IR (ATR-Ge, cm^{-1}): 3267 (ν, NH), 1694 (ν, C=O, amide), 1576, 1512; Calculated for $C_{13}H_{10}Cl_2N_2O$ (MW 281.13): 55.54% C; 3.58% H; 9.97% N; found 55.25% C; 3.18% H; 9.96% N; CAS Registry Number 522619-43-8.

2-(2-chlorophenyl)-*N*-(6-chloropyridin-2-yl)acetamide (**15**)

mp. 99.2–102.9 °C; Yield 18%; ^{1}H NMR (600 MHz, DMSO) δ 11.03 (s, 1H, CONH), 7.98 (d, *J* = 8.3 Hz, 1H, ArH), 7.78 (t, *J* = 8.0 Hz, 1H, ArH), 7.44–7.34 (m, 2H, ArH), 7.30–7.18 (m, 2H, ArH), 7.16 (d, *J* = 7.5 Hz, 1H, ArH), 3.88 (s, 2H, CH_2); ^{13}C NMR (151 MHz, DMSO) δ 169.68, 152.57, 148.56, 142.31, 134.28, 134.04, 132.87, 129.55, 129.26, 127.62, 119.60, 112.43, 41.13; IR (ATR-Ge, cm^{-1}): 3302 (ν, NH), 1675 (ν, C=O, amide), 1589, 1571, 1517; Calculated for $C_{13}H_{10}Cl_2N_2O$ (MW 281.13): 55.54% C; 3.58% H; 9.97% N; found 55.90% C; 3.48% H; 9.68% N; CAS Registry Number 1710692-93-5.

2-(3-chlorophenyl)-*N*-(pyridin-2-yl)acetamide (**16**)

mp. 100.2–102.1 °C; Yield 53%; ^{1}H NMR (600 MHz, DMSO) δ 10.69 (s, 1H, CONH), 8.31–8.24 (m, 1H, ArH), 8.00 (d, *J* = 8.5 Hz, 1H, ArH), 7.76–7.67 (m, 1H, ArH), 7.40–7.34 (m, 1H, ArH), 7.35–7.23 (m, 3H, ArH), 7.08–7.01 (m, 1H, ArH), 3.71 (s, 2H, CH_2); ^{13}C NMR (151 MHz, DMSO) δ 169.99, 152.48, 148.52, 138.73, 133.36, 130.67, 129.66, 128.52, 127.15, 120.04, 113.95, 42.92; IR (ATR-Ge, cm^{-1}): 3240 (ν, NH), 1662 (ν, C=O, amide), 1597, 1577, 1539; Calculated for $C_{13}H_{11}ClN_2O$ (MW 246.68): 63.29% C; 4.49% H; 11.36% N; found 62.89% C; 4.18% H; 11.12% N; CAS Registry Number 1385550-70-8.

2-(3-chlorophenyl)-*N*-(5-chloropyridin-2-yl)acetamide (**17**)

mp. 110.5–112.4 °C; Yield 11%; ^{1}H NMR (600 MHz, DMSO) δ 10.88 (s, 1H, CONH), 8.36–8.31 (m, 1H, ArH), 8.04 (d, J = 8.9 Hz, 1H, ArH), 7.87–7.80 (m, 1H, ArH), 7.39–7.33 (m, 1H, ArH), 7.35–7.22 (m, 3H, ArH), 3.72 (s, 2H, CH_2); ^{13}C NMR (151 MHz, DMSO) δ 170.13, 151.13, 146.90, 138.47, 133.37, 130.68, 129.69, 128.55, 127.19, 125.70, 115.04, 42.83; IR (ATR-Ge, cm^{-1}): 3447 (ν, NH), 1664 (ν, C=O, amide), 1574, 1559, 1550; Calculated for $C_{13}H_{10}Cl_2N_2O$ (MW 281.13): 55.54% C; 3.58% H; 9.97% N; found 55.89% C; 3.44% H; 9.78% N; CAS Registry Number 2200680-24-4.

2-(3-chlorophenyl)-*N*-(6-chloropyridin-2-yl)acetamide (**18**)

mp. 97.4–98.7 °C; Yield 20%; ^{1}H NMR (500 MHz, DMSO) δ 11.04 (s, 1H, CONH), 8.02 (d, *J* = 8.2 Hz, 1H, ArH), 7.82 (t, *J* = 8.0 Hz, 1H, ArH), 7.43–7.39 (m, 1H, ArH), 7.38–7.26 (m, 3H, ArH), 7.22–7.16 (m, 1H, ArH), 3.74 (s, 2H, CH_2); ^{13}C NMR (126 MHz, DMSO) δ 170.17, 152.41, 148.46, 142.25, 138.29, 133.30, 130.62, 129.62, 128.47, 127.15, 119.66, 112.41, 42.78; IR (ATR-Ge, cm^{-1}): 3251 (ν, NH), 1668 (ν, C=O, amide), 1591, 1571, 1535; Calculated for $C_{13}H_{10}Cl_2N_2O$ (MW 281.13): 55.54% C; 3.58% H; 9.97% N; found 55.22% C; 3.44% H; 9.85% N; CAS Registry Number 1713578-95-0.

2-(4-chlorophenyl)-*N*-(pyridin-2-yl)acetamide (**19**)

mp. 123.0–124.5 °C; Yield 27%; ^{1}H NMR (500 MHz, $CDCl_3$) δ 8.37 (s, 1H, CONH), 8.26–8.18 (m, 2H, ArH), 7.74–7.67 (m, 1H, ArH), 7.38 –7.28 (m, 2H, ArH), 7.28–7.18 (m, 2H, ArH), 7.09–7.00 (m, 1H, ArH), 3.72 (s, 2H, CH_2); ^{13}C NMR (126 MHz, $CDCl_3$) δ 168.97, 151.16, 147.59, 138.52, 133.59, 132.40, 130.72, 129.20, 120.00, 114.12, 43.99; IR (ATR-Ge, cm^{-1}): 3259 (ν, NH), 1695 (ν, C=O, amide), 1597, 1582, 1532; Calculated for $C_{13}H_{11}ClN_2O$ (MW 246.68): 63.29% C; 4.49% H; 11.36% N; found 62.93% C; 4.12% H; 11.00% N; CAS Registry Number 349429-86-3.

2-(4-chlorophenyl)-*N*-(5-chloropyridin-2-yl)acetamide (**20**)

mp. 145.0–145.5 °C; Yield 80%; ^{1}H NMR (600 MHz, $CDCl_3$) δ 8.17 (d, *J* = 8.8 Hz, 1H, ArH), 8.15 (d, *J* = 2.6 Hz, 1H, ArH), 7.99 (s, 1H, CONH), 7.66–7.61 (m, 1H, ArH), 7.36–7.31 (m, 2H, ArH), 7.26–7.21 (m, 2H, ArH), 3.70 (s, 2H, CH_2); ^{13}C NMR (151 MHz, $CDCl_3$) δ 168.94, 149.45, 146.49, 138.17, 133.92, 132.21, 130.86, 129.45, 127.15, 114.74, 44.10; IR (ATR-Ge, cm^{-1}): 3245 (ν, NH), 1704 (ν, C=O, amide), 1557, 1523, 1490; Calculated for $C_{13}H_{10}Cl_2N_2O$ (MW 281.13): 55.54% C; 3.58% H; 9.97% N; found 55.83% C; 3.23% H; 9.88% N; CAS Registry Number 349418-27-5.

2-(4-chlorophenyl)-*N*-(6-chloropyridin-2-yl)acetamide (**21**)

mp. 121.0–122.0 °C; Yield 55%; ^{1}H NMR (500 MHz, $CDCl_3$) δ 8.14 (d, *J* = 8.2 Hz, 1H, ArH), 7.95 (s, 1H, CONH), 7.66 (t, *J* = 7.9 Hz, 1H, ArH), 7.39–7.32 (m, 2H, ArH), 7.29–7.22 (m, 2H, ArH), 7.09–7.04 (m, 1H, ArH), 3.72 (s, 2H, CH_2); ^{13}C NMR (126 MHz, $CDCl_3$) δ 168.95, 150.83, 148.89, 140.97, 133.80, 131.92, 130.72, 129.32, 119.98, 112.00, 44.05; IR (ATR-Ge, cm^{-1}): 3273 (ν, NH), 1673 (ν, C=O, amide), 1589, 1573, 1529; Calculated for $C_{13}H_{10}Cl_2N_2O$ (MW 281.13): 55.54% C; 3.58% H; 9.97% N; found 55.76% C; 3.46% H; 9.88% N; CAS Registry Number 1710380-48-5.

N-(pyridin-2-yl)-2-[2-(trifluoromethyl)phenyl]acetamide (**22**)

mp. 82.3–84.6 °C; Yield 46%; ^{1}H NMR (600 MHz, DMSO) δ 10.70 (s, 1H, CONH), 8.32–8.26 (m, 1H, ArH), 7.97 (d, J = 8.4 Hz, 1H, ArH), 7.74–7.68 (m, 1H, ArH), 7.66 (d, *J* = 7.8 Hz, 1H, ArH), 7.60 (t, *J* = 7.6 Hz, 1H, ArH), 7.50–7.40 (m, 2H, ArH), 7.08–7.01 (m, 1H, ArH), 3.98 (s, 2H, CH_2); ^{13}C NMR (151 MHz, DMSO) δ 169.55, 152.56, 148.52, 138.71, 134.29, 134.08, 132.77, 128.20 (q, *J* = 29.0 Hz), 127.88, 126.17 (q, *J* = 5.4 Hz), 125.02 (q, *J* = 274.2 Hz), 119.91, 113.87, 40.16; IR (ATR-Ge, cm^{-1}): 3328 (ν, NH), 1681 (ν, C=O, amide), 1595, 1577.

N-(5-chloropyridin-2-yl)-2-[2-(trifluoromethyl)phenyl]acetamide (**23**)

mp. 115.4–116.8 °C; Yield 15%; ^{1}H NMR (500 MHz, DMSO) δ 10.96 (s, 1H, CONH), 8.39–8.35 (m, 1H, ArH), 8.08 (d, *J* = 8.9 Hz, 1H, ArH), 7.87 (dd, *J* = 8.9, 2.7 Hz, 1H, ArH), 7.71 (s, 1H, ArH), 7.66–7.59 (m, 2H, ArH), 7.56 (t, *J* = 7.7 Hz, 1H, ArH), 3.86 (s, 2H, CH_2); ^{13}C NMR (151 MHz, DMSO) δ 169.71, 151.19, 146.88, 140.89, 138.47, 134.06, 132.79, 130.66, 128.21 (q, *J* =. 30.1 Hz), 126.19 (q, *J* = 5.9 Hz), 125.65 (*J* = 5.4 Hz), 125.00 (q, *J* = 273.3 Hz), 114.95, 40.13; IR (ATR-Ge, cm^{-1}): 3325 (ν, NH), 1680 (ν, C=O,

amide), 1592, 1574, 1518; HRMS (ESI$^+$): $[M+H]^+$ calcd. for $C_{14}H_{11}ClF_3N_2O^+$ (m/z): 315.05065, found 315.05029.

N-(6-chloropyridin-2-yl)-2-[2-(trifluoromethyl)phenyl]acetamide (**24**)

mp. 117.3–118.2 °C; Yield 57%; ^{1}H NMR (600 MHz, DMSO) δ 10.90 (s, 1H, CONH), 8.34 (d, *J* = 2.7 Hz, 1H, ArH), 8.05–7.96 (m, 1H, ArH), 7.87–7.78 (m, 1H, ArH), 7.71–7.62 (m, 1H, ArH), 7.64–7.55 (m, 1H, ArH), 7.52–7.39 (m, 2H, ArH), 3.99 (s, 2H, CH_2); ^{13}C NMR (151 MHz, DMSO) δ 169.62, 151.11, 146.82, 138.39, 134.02, 133.97 (q, *J* = 4.9 Hz), 132.72, 128.20 (q, *J* = 29.5 Hz), 127.87, 125.50, 126.19 (q, *J* = 5.7 Hz), 125.00 (q, *J* = 273.8 Hz), 114.87, 40.12; IR (ATR-Ge, cm^{-1}): 3326 (ν, NH), 1680 (ν, C=O, amide), 1594, 1572, 1519; HPLC purity: 98.35%; HRMS (ESI$^+$): $[M+H]^+$ calcd. for $C_{14}H_{11}ClF_3N_2O^+$ (m/z): 315.05065, found 315.05023.

N-(pyridin-2-yl)-2-[3-(trifluoromethyl)phenyl]acetamide (**25**)

mp. 79.6–81.7 °C; Yield 55%; ^{1}H NMR (600 MHz, DMSO) δ 10.76 (s, 1H, CONH), 8.30–8.24 (m, 1H, ArH), 8.00 (d, *J* = 8.4 Hz, 1H, ArH), 7.75–7.69 (m, 1H, ArH), 7.68 (d, *J* = 1.9 Hz, 1H, ArH), 7.64–7.55 (m, 2H, ArH), 7.55–7.48 (m, 1H, ArH), 7.09–7.01 (m, 1H, ArH), 3.82 (s, 2H, CH_2); ^{13}C NMR (151 MHz, DMSO) δ 169.98, 152.48, 148.52, 138.72, 137.67, 134.01, 129.85, 129.42 (q, *J* = 31.6 Hz), 126.40 (q, J = 3.9 Hz),124.79 (q, *J* = 276.6 Hz), 123.29 (q, *J* = 4.1 Hz), 120.05, 113.97, 42.93; IR (ATR-Ge, cm^{-1}): 3253 (ν, NH), 1702 (ν, C=O, amide), 1584, 1559; HPLC purity: 97.53%; HRMS (ESI$^+$): $[M+H]^+$ calcd. for $C_{14}H_{12}F_3N_2O^+$ (m/z): 281.08962, found 281.08914; CAS Registry Number 1330923-98-2.

N-(5-chloropyridin-2-yl)-2-[3-(trifluoromethyl)phenyl]acetamide (**26**)

mp. 126.4–127.8 °C; Yield 40%; ^{1}H NMR (600 MHz, DMSO) δ 10.93 (s, 1H, CONH), 8.33 (d, *J* = 2.6 Hz, 1H, ArH), 8.04 (d, *J* = 8.9 Hz, 1H, ArH), 7.86–7.81 (m, 1H, ArH), 7.67 (s, 1H, ArH), 7.63–7.55 (m, 2H, ArH), 7.53 (m, 1H, ArH), 3.83 (s, 2H, CH_2); ^{13}C NMR (151 MHz, DMSO) δ 169.63, 151.11, 146.83 (q, *J* = 7.7 Hz), 138.40, 134.08, 134.03, 132.71, 128.19 (q, *J* = 29.7 Hz), 127.87, 126.17 (q, *J* = 5.7 Hz), 125.50, 124.77 (q, *J* = 273.7 Hz), 114.87, 40.51; IR (ATR-Ge, cm^{-1}): 3232 (ν, NH), 1658 (ν, C=O, amide), 1592, 1574, 1524; HRMS (ESI$^+$): $[M+H]^+$ calcd. for $C_{14}H_{11}ClF_3N_2O^+$ (m/z): 315.05065, found 315.05029.

N-(6-chloropyridin-2-yl)-2-[3-(trifluoromethyl)phenyl]acetamide (**27**)

mp. 85.4–86.5 °C; Yield 47%; ^{1}H NMR (600 MHz, DMSO) δ 11.05 (s, 1H, CONH), 7.99 (d, J = 8.0 Hz, 1H, ArH), 7.78 (t, J = 7.9 Hz, 1H, ArH), 7.67 (d, J = 2.1 Hz, 1H, ArH), 7.62 – 7.55 (m, 2H, ArH), 7.52 (t, J = 7.7 Hz, 1H, ArH), 7.15 (d, J = 7.7 Hz, 1H, ArH), 3.82 (s, 2H, CH_2); ^{13}C NMR (151 MHz, DMSO) δ 170.23, 152.48, 148.55, 142.32, 137.32, 134.04, 129.86, 129.56 (q, *J* = 31.7 Hz), 126.47 (q, *J* = 3.1 Hz),124.79 (q, *J* = 272.9 Hz), 124.01 (q, *J* = 3.1 Hz), 119.75, 112.51, 42.87; IR (ATR-Ge, cm^{-1}): 3245 (ν, NH), 1666 (ν, C=O, amide), 1594, 1572, 1559; HPLC purity: 99.89%; HRMS (ESI$^+$): $[M+H]^+$ calcd. for $C_{14}H_{11}ClF_3N_2O^+$ (m/z): 315.05065, found 315.05032; CAS Registry Number 1265538-65-5.

N-(pyridin-2-yl)-2-[4-(trifluoromethyl)phenyl]acetamide (**28**)

mp. 139.2–141.7 °C; Yield 61%; ^{1}H NMR (600 MHz, DMSO) δ 10.75 (s, 1H, CONH), 8.30–8.24 (m, 1H, ArH), 8.00 (d, *J* = 8.3 Hz, 1H, ArH), 7.76 –7.68 (m, 1H, ArH), 7.65 (d, *J* = 8.1 Hz, 2H, ArH), 7.53 (d, *J* = 8.1 Hz, 2H, ArH), 7.08–7.01 (m, 1H, ArH), 3.81 (s, 2H, CH_2); ^{13}C NMR (151 MHz, DMSO) δ 169.84, 152.47, 148.52, 141.14, 138.73, 130.64, 127.92 (q, *J* = 31.1 Hz), 125.66 (q, *J* = 4.3 Hz), 124.92 (q, *J* = 271.7 Hz), 120.06, 113.95, 43.13; IR (ATR-Ge, cm^{-1}): 3253 (ν, NH), 1692 (ν, C=O, amide), 1619, 1598, 1586; HPLC purity: 99.68%; HRMS (ESI$^+$): $[M+H]^+$ calcd. for $C_{14}H_{12}F_3N_2O^+$ (m/z): 281.08962, found 281.08929; CAS Registry Number 1326205-29-1.

N-(5-chloropyridin-2-yl)-2-[4-(trifluoromethyl)phenyl]acetamide (**29**)

mp. 164.5–167.8 °C; Yield 33%; ^{1}H NMR (600 MHz, DMSO) δ 10.94 (s, 1H, CONH), 8.33 (d, *J* = 2.6 Hz, 1H, ArH), 8.04 (d, *J* = 8.9 Hz, 1H, ArH), 7.87 – 7.81 (m, 1H, ArH), 7.65 (d, *J* = 8.0 Hz, 2H, ArH), 7.52 (d, *J* = 8.0 Hz, 2H, ArH), 3.82 (s, 2H, CH_2); ^{13}C NMR (151 MHz, DMSO) δ 169.99, 151.11, 146.90, 140.91, 138.48, 130.97, 130.68, 127.96 (q, *J* = 31.8 Hz), 125.68 (q, *J* = 3.4 Hz), 124.90 (q, *J* = 272.7 Hz), 115.04, 43.05; IR (ATR-Ge, cm^{-1}): 3233 (ν, NH), 1660 (ν, C=O, amide), 1592, 1573, 1532; HPLC purity: 94.35%; HRMS (ESI^{+}): [M+H]$^{+}$ calcd. for $C_{14}H_{11}ClF_3N_2O^{+}$ (m/z): 315.05065, found 315.05026; CAS Registry Number 1099892-28-0.

N-(6-chloropyridin-2-yl)-2-[4-(trifluoromethyl)phenyl]acetamide (**30**)

mp. 114.7–115.7 °C; Yield 52%; ^{1}H NMR (600 MHz, DMSO) δ 11.06 (s, 1H, CONH), 7.98 (d, *J* = 8.2 Hz, 1H, ArH), 7.78 (t, *J* = 8.0 Hz, 1H, ArH), 7.65 (d, *J* = 8.0 Hz, 2H, ArH), 7.52 (d, *J* = 7.9 Hz, 2H, ArH), 7.16 (d, *J* = 7.6 Hz, 1H, ArH), 3.81 (s, 2H, CH_2); ^{13}C NMR (151 MHz, DMSO) δ 170.10, 152.46, 148.55, 142.33, 140.79, 130.68, 127.99 (q, *J* = 31.6 Hz),125.69 (q, *J* = 3.8 Hz), 124.89 (q, *J* = 272.1 Hz), 119.77, 112.49, 43.06; IR (ATR-Ge, cm^{-1}): 3233 (ν, NH), 1661 (ν, C=O, amide), 1595, 1571, 1543; HPLC purity: 99.34%; HRMS (ESI^{+}): [M+H]$^{+}$ calcd. for $C_{14}H_{11}ClF_3N_2O^{+}$ (m/z): 315.05065, found 315.05029; CAS Registry Number 1713453-76-9.

2-(4-fluorophenyl)-*N*-(pyridin-2-yl)acetamide (**31**)

mp. 134–136 °C; Yield 48%; ^{1}H NMR (500 MHz, $CDCl_3$) δ 8.33 (s, 1H, CONH), 8.25–8.20 (m, 2H, ArH), 7.74–7.67 (m, 1H, ArH), 7.39 –7.25 (m, 2H, ArH), 7.14–6.98 (m, 3H, ArH), 3.73 (s, 2H, CH_2); ^{13}C NMR (126 MHz, $CDCl_3$) δ 169.30, 162.25 (d, *J* = 246.4 Hz), 151.17, 147.54, 138.54, 131.01 (d, *J* = 8.1 Hz), 129.71 (d, *J* = 3.3 Hz), 119.96, 115.9 (d, *J* = 21.7 Hz) , 114.07, 43.87; IR (ATR-Ge, cm^{-1}): 3237 (ν, NH), 1661 (ν, C=O, amide), 1579, 1547, 1538; HPLC purity: 99.38%; HRMS (ESI^{+}): [M+H]$^{+}$ calcd. for $C_{13}H_{12}FN_2O^{+}$ (m/z): 231.09282, found 231.09247; CAS Registry Number 432536-56-6.

2-(4-fluorophenyl)-*N*-(5-chloropyridin-2-yl)acetamide (**32**)

mp. 146.5–148.0 °C; Yield 67%; ^{1}H NMR (500 MHz, DMSO) δ 11.03 (s, 1H, CONH), 8.02 (d, *J* = 8.2 Hz, 1H, ArH), 7.81 (t, *J* = 7.9 Hz, 1H, ArH), 7.39–7.32 (m, 2H, ArH), 7.22–7.16 (m, 1H, ArH), 7.18–7.08 (m, 2H, ArH), 3.70 (s, 2H, CH_2); ^{13}C NMR (126 MHz, $CDCl_3$) δ 169.17, 162.31 (d, *J* = 247.0 Hz), 149.37, 146.34, 138.03, 131.03 (d, *J* = 8.1 Hz), 129.41 (d, *J* = 3.4 Hz), 126.98, 116.11 (d, *J* = 21.5 Hz), 114.60, 43.84; IR (ATR-Ge, cm^{-1}): 3257 (ν, NH), 1694 (ν, C=O, amide), 1602, 1589, 1578; HPLC purity: 99.01%; HRMS (ESI^{+}): [M+H]$^{+}$ calcd. for $C_{13}H_{11}ClFN_2O^{+}$ (m/z): 265.05385, found 265.05371; CAS Registry Number 432515-15-6.

2-(4-fluorophenyl)-*N*-(6-chloropyridin-2-yl)acetamide (**33**)

mp. 119.2–120.3 °C; Yield 25%; ^{1}H NMR (600 MHz, DMSO) δ 10.85 (s, 1H, CONH), 8.34–8.31 (m, 1H, ArH), 8.04 (d, J = 8.9 Hz, 1H, ArH), 7.86–7.79 (m, 1H, ArH), 7.37–7.29 (m, 2H, ArH), 7.14–7.04 (m, 2H, ArH), 3.68 (s, 2H, CH_2); ^{13}C NMR (126 MHz, $CDCl_3$) δ 169.29, 162.32 (d, *J* = 246.9 Hz), 150.88, 148.88, 140.95, 131.01 (d, *J* = 8.1 Hz), 129.24 (d, *J* = 3.3 Hz), 119.93, 116.11 (d, *J* = 21.7 Hz), 111.98, 43.91; IR (ATR-Ge, cm^{-1}): 3990 (ν, NH), 1702 (ν, C=O, amide), 1570, 1509, 1433; HPLC purity: 96.68%; HRMS (ESI^{+}): [M+H]$^{+}$ calcd. for $C_{13}H_{11}ClFN_2O^{+}$ (m/z): 265.05385, found 265.05347; CAS Registry Number 1710330-25-8.

1.8. ^{1}H NMR and ^{13}C NMR spectra of selected compounds

(Compounds **12**, **17**, **21**, **24**, **26**)

SEE THE NEXT PAGE…

Compound 12

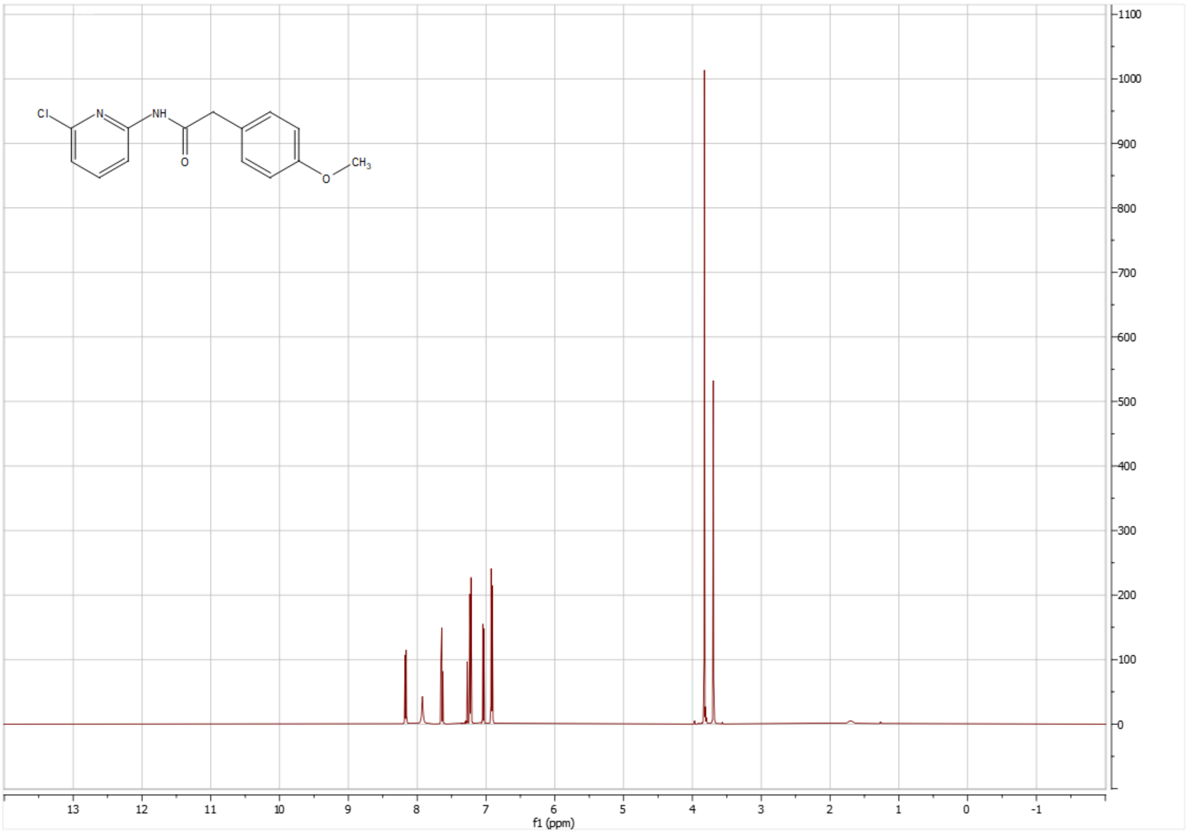

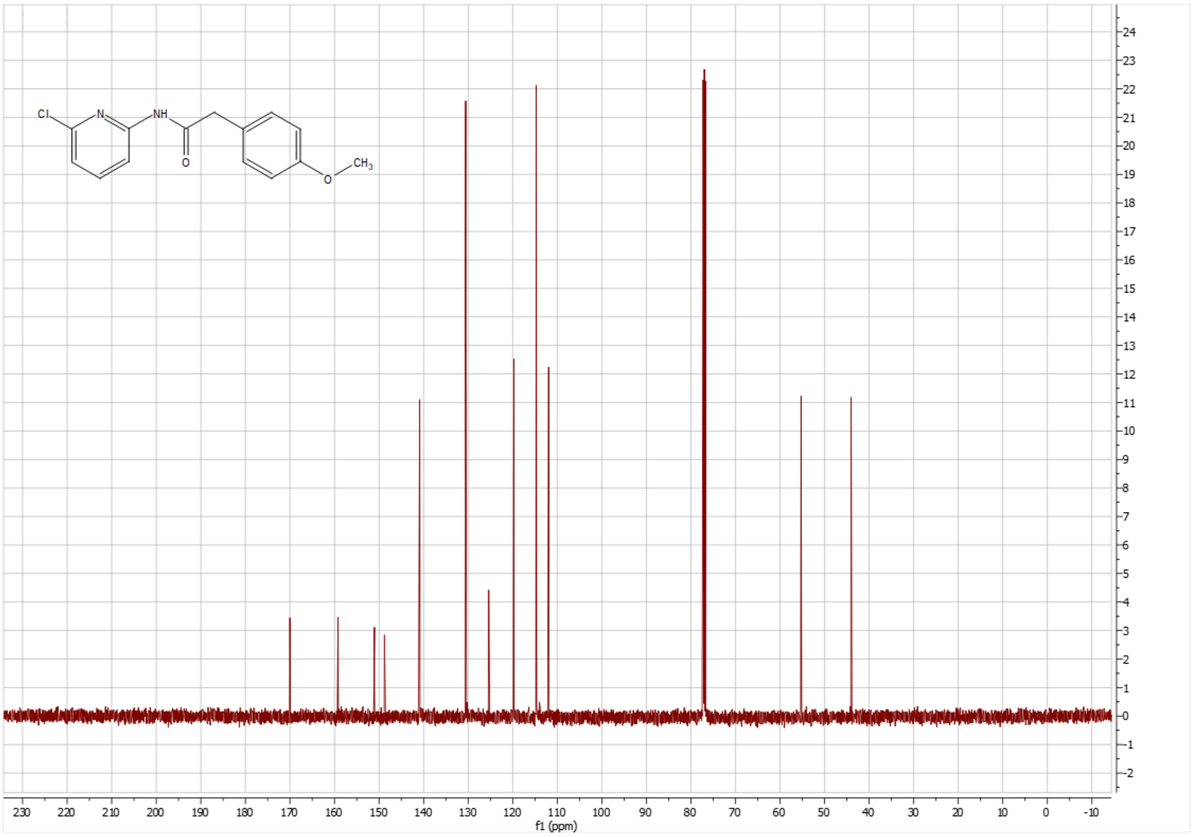

Compound 17

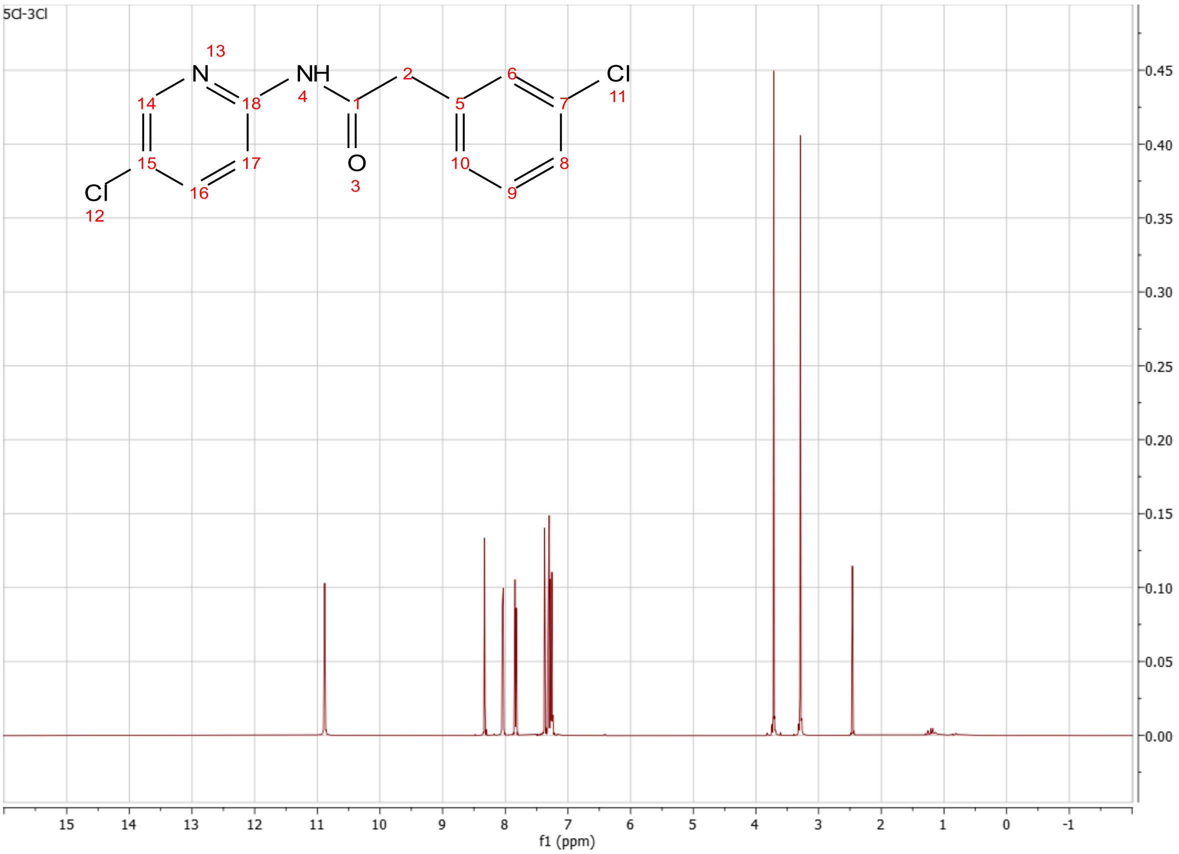
5Cl-3Cl
N
NH
Cl
O
Cl
13
14
18
4
1
2
6
7
11
5
15
17
10
8
16
3
9
12
0.45
0.40
0.35
0.30
0.25
0.20
0.15
0.10
0.05
0.00
15
14
13
12
11
10
9
8
7
6
5
4
3
2
1
0
-1
f1 (ppm)

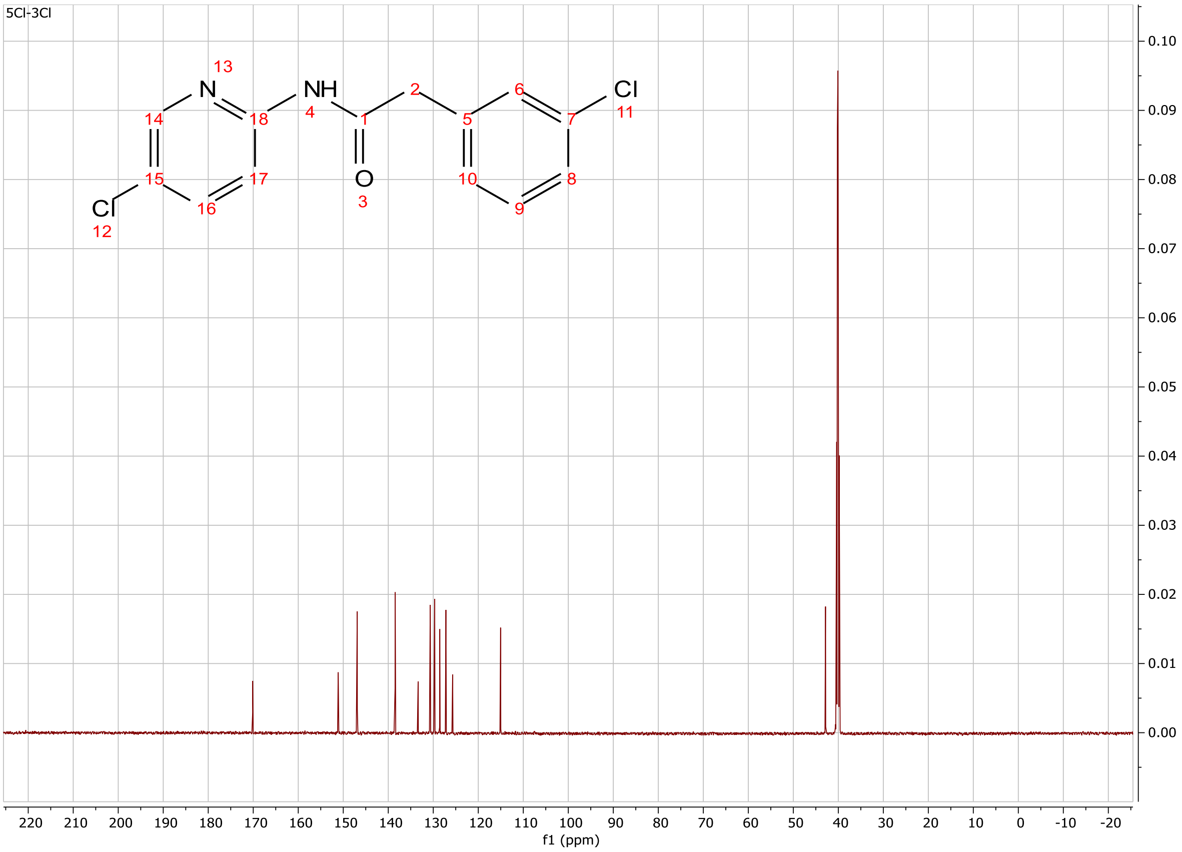
5Cl-3Cl
N
NH
Cl
O
Cl
13
14
18
4
1
2
6
7
11
5
15
17
10
8
16
3
9
12
0.10
0.09
0.08
0.07
0.06
0.05
0.04
0.03
0.02
0.01
0.00
220
210
200
190
180
170
160
150
140
130
120
110
100
90
80
70
60
50
40
30
20
10
0
-10
-20
f1 (ppm)

Compound 21

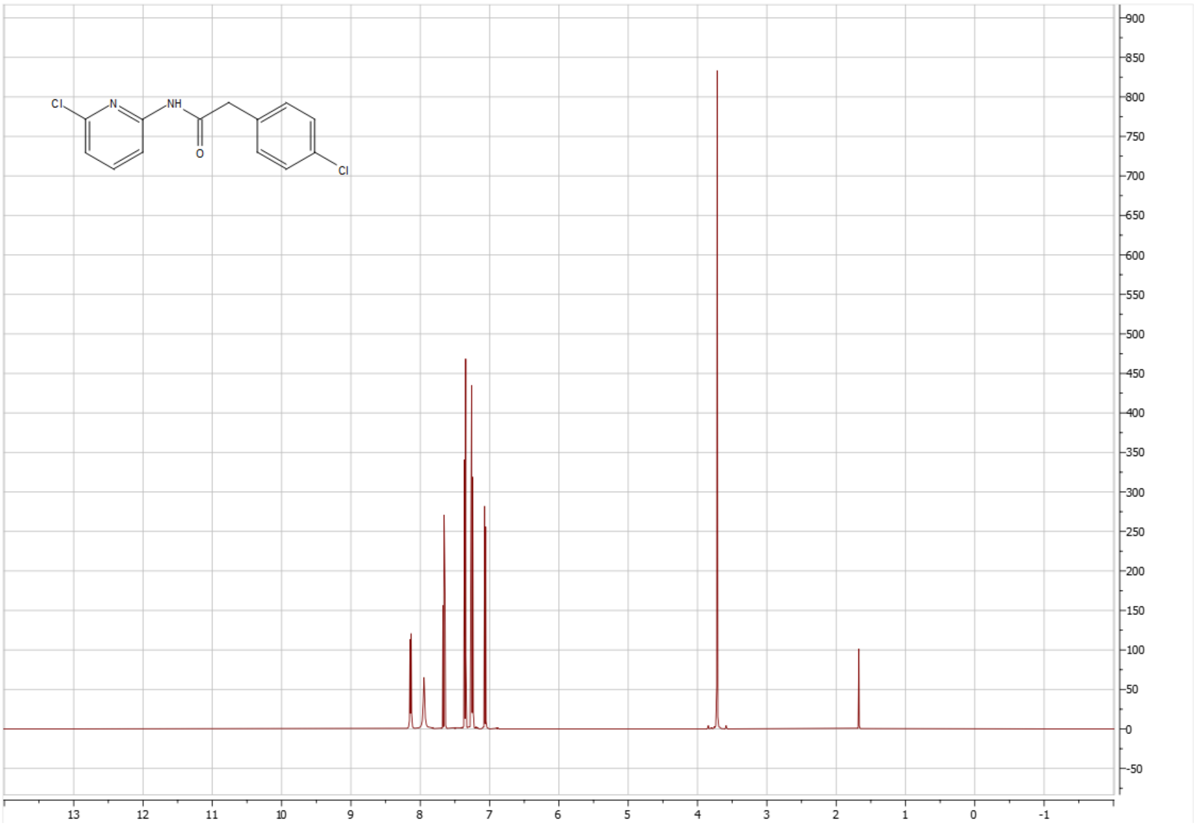
Cl
N
NH
O
Cl
900
850
800
750
700
650
600
550
500
450
400
350
300
250
200
150
100
50
0
-50
13
12
11
10
9
8
7
6
5
4
3
2
1
0
-1

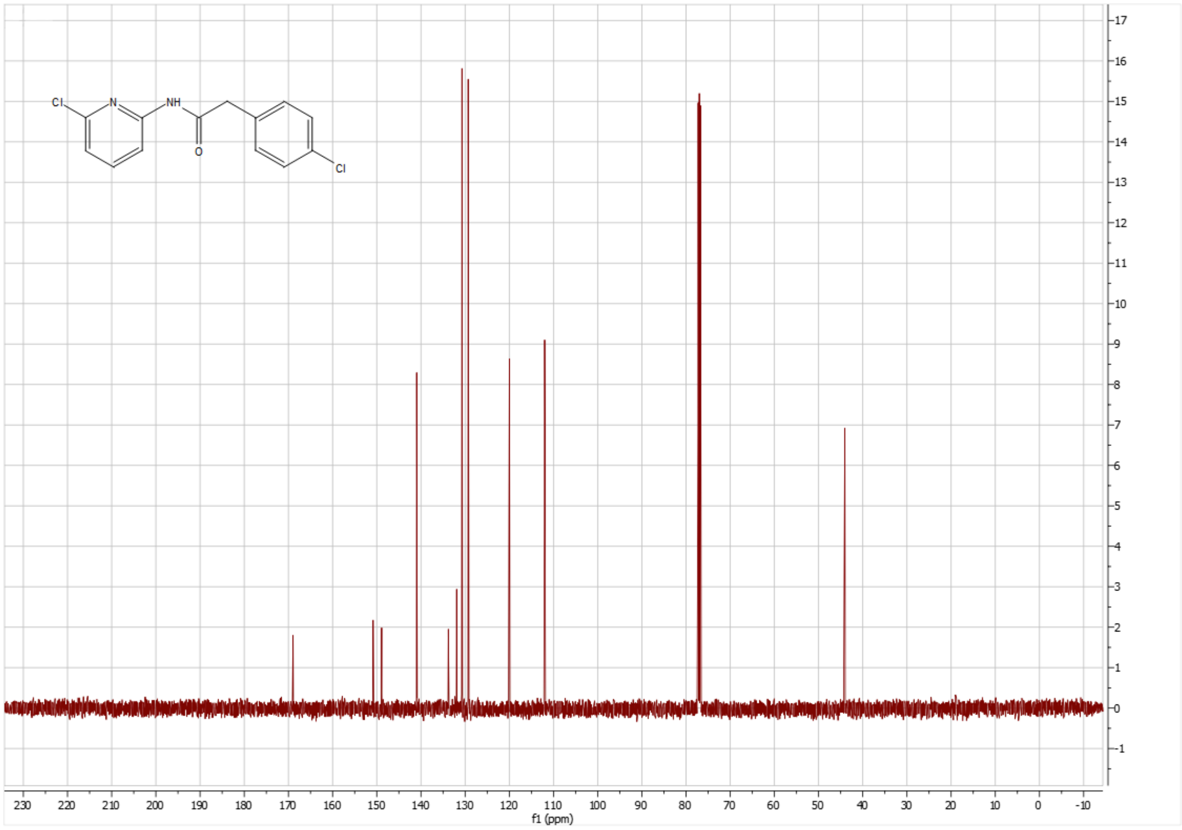
Cl
N
NH
O
Cl
17
16
15
14
13
12
11
10
9
8
7
6
5
4
3
2
1
0
-1
230
220
210
200
190
180
170
160
150
140
130
120
110
100
90
80
70
60
50
40
30
20
10
0
-10
f1 (ppm)

Compound 24

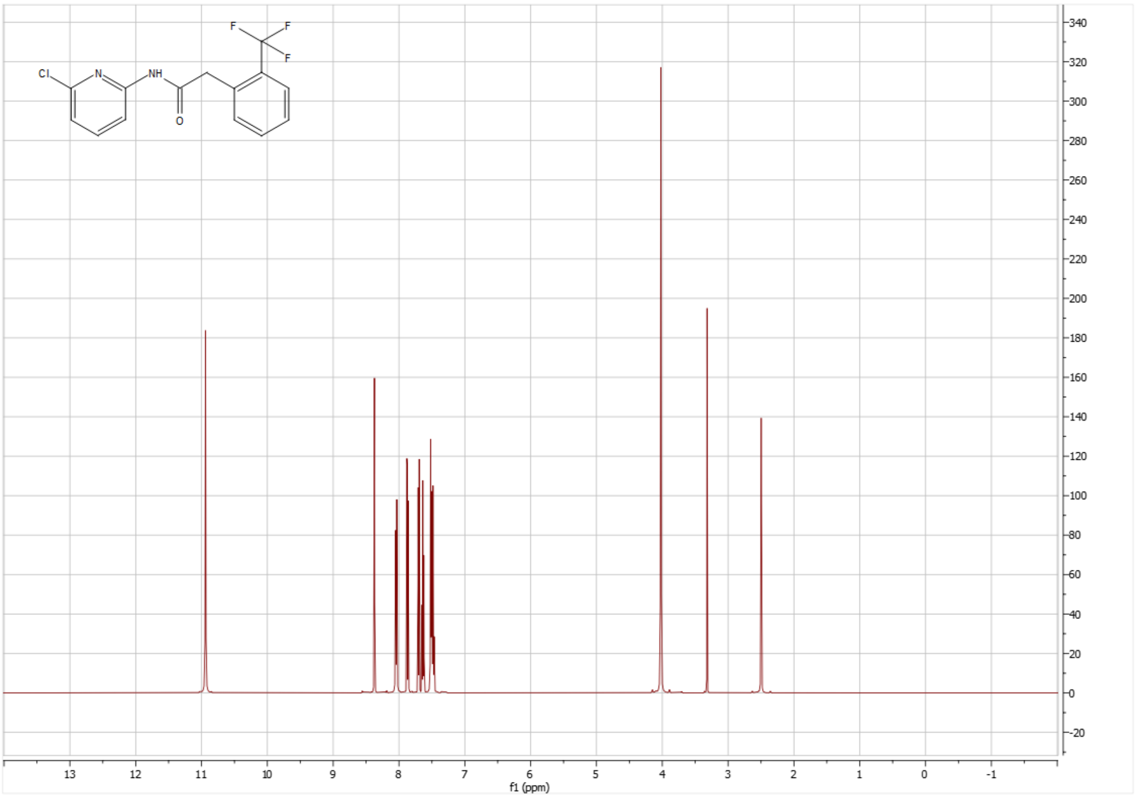

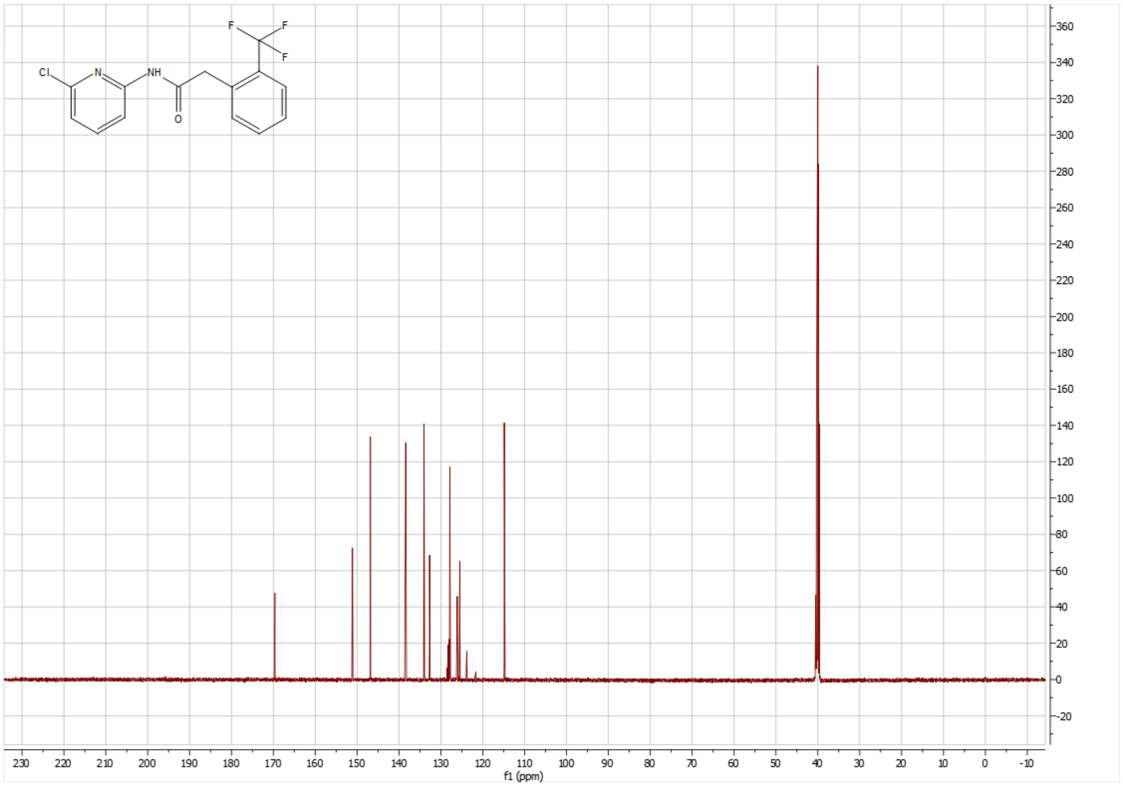

Compound 26

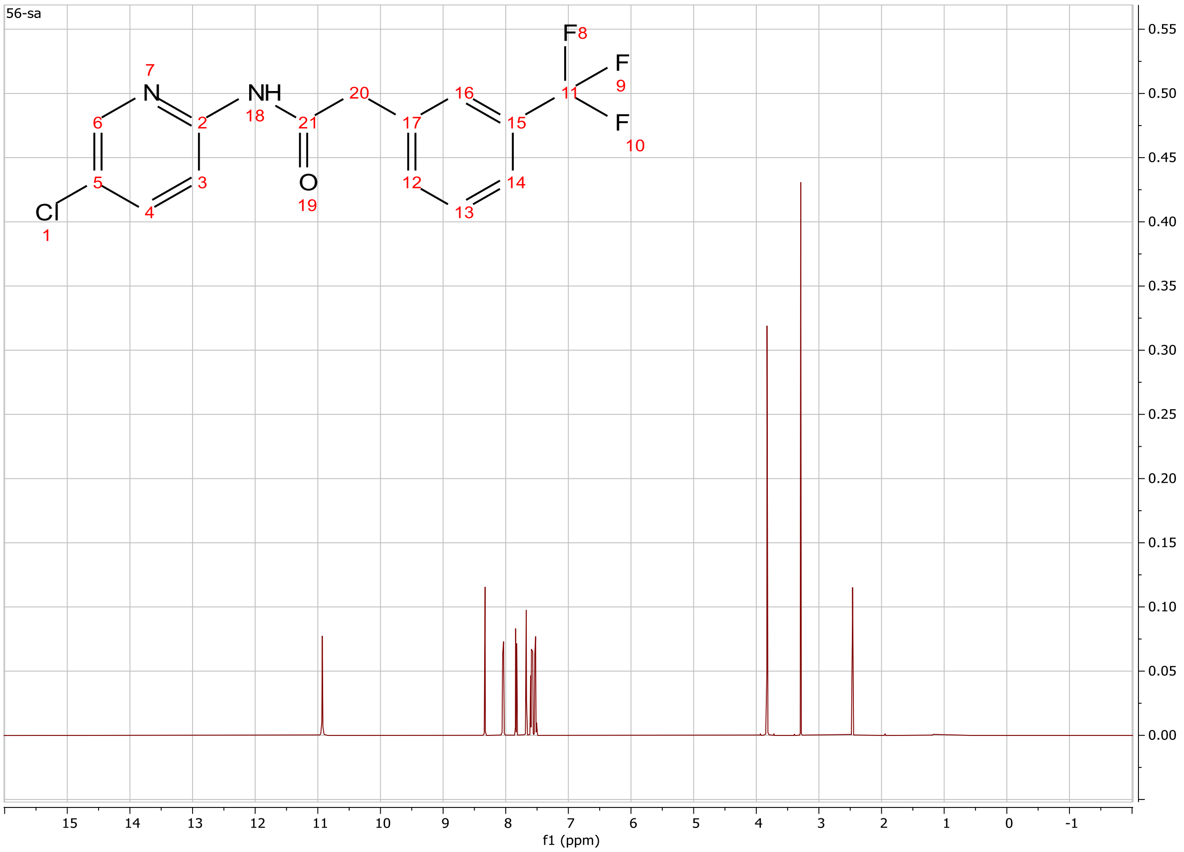

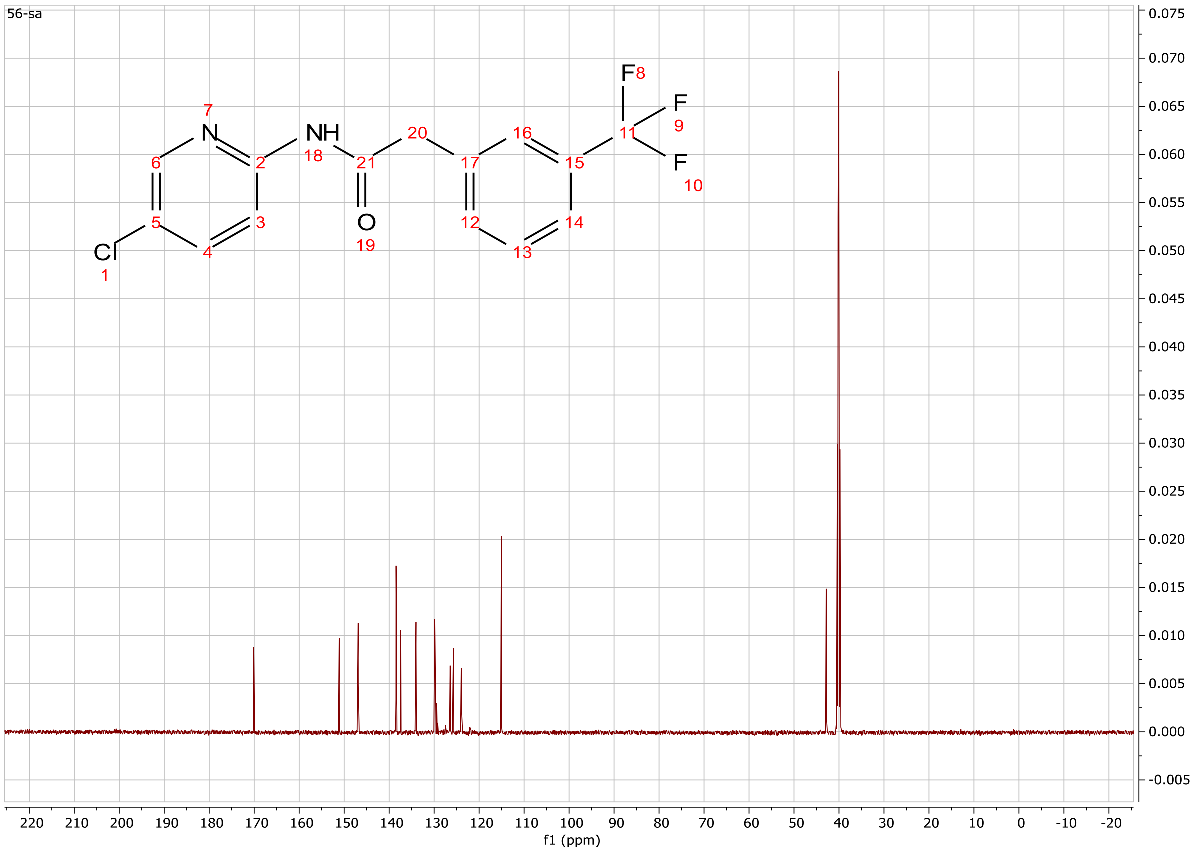

1.9. References

1. Schön T, Werngren J, Machado D, et al. Antimicrobial susceptibility testing of Mycobacterium tuberculosis complex isolates - the EUCAST broth microdilution reference method for MIC determination. *Clin Microbiol Infect Off Publ Eur Soc Clin Microbiol Infect Dis*. 2020;26(11):1488-1492. doi: 10.1016/j.cmi.2020.07.036

2. Franzblau SG, Witzig RS, McLaughlin JC, et al. Rapid, low-technology MIC determination with clinical Mycobacterium tuberculosis isolates by using the microplate Alamar Blue assay. *J Clin Microbiol*. 1998;36(2):362-366. doi: 10.1128/JCM.36.2.362-366.1998

3. European Committee for Antimicrobial Susceptibility Testing (EUCAST) of the European Society of Clinical Microbiology and Infectious Diseases (ESCMID). Determination of minimum inhibitory concentrations (MICs) of antibacterial agents by broth dilution. *Clin Microbiol Infect*. 2003;9(8):ix-xv. doi: 10.1046/j.1469-0691.2003.00790.x

4. Arendrup MC, Cuenca-Estrella M, Lass-Flörl C, Hope W. EUCAST-AFST. EUCAST technical note on the EUCAST definitive document EDef 7.2: method for the determination of broth dilution minimum inhibitory concentrations of antifungal agents for yeasts EDef 7.2 (EUCAST-AFST). *Clin Microbiol Infect Off Publ Eur Soc Clin Microbiol Infect Dis*. 2012;18(7):E246-247. doi: 10.1111/j.1469-0691.2012.03880.x

5. Arendrup MC, Meletiadis J, Mouton JW, Lagrou K, Hamal P, Guinea J. Subcommittee on Antifungal Susceptibility Testing of the ESCMID European Committee for Antimicrobial Susceptibility Testing . 2017. EUCAST definitive document E.DEF 7.3.1. Method for the determination of broth dilution minimum inhibitory concentrations of antifungal agents for yeasts. http://www.eucast.org/fileadmin/src/media/PDFs/EUCAST_files/AFST/Files/EUCAST_E_Def_7_3_1_Yeast_testing__definitive.pdf

6. Finger V, Kucera T, Kafkova R, et al. 2,6-Disubstituted 7-(naphthalen-2-ylmethyl)-7H-purines as a new class of potent antitubercular agents inhibiting DprE1. Eur. J. Med. Chem. 2023;258: 115611. doi: 10.1016/j.ejmech.2023.115611
